# In-depth behavioral characterization of a rat model of Schaaf-Yang syndrome

**DOI:** 10.1038/s41598-025-20958-y

**Published:** 2025-10-30

**Authors:** Felix Franke, Semih Ertürk, Johann G. Maass, Dominik Kamionek, Tim Schubert, Claudia Pitzer, Susanne Theiß, Christine Fischer, Rachel B. Gilmore, Eva Dwornicki, Colleen R. Bocke, Gina L. C. Yosten, Christian P. Schaaf, Ferdinand Althammer

**Affiliations:** 1 Institute of Human Genetics, Heidelberg University, Heidelberg, Germany; 2 Division of Genetics and Genomics, Boston Children’s Hospital, Boston, MA USA; 3 Department of Applied Mathematics and Theoretical Physics, Cambridge University, Cambridge, UK; 4 Interdisciplinary Neurobehavioral Core, Heidelberg University, Heidelberg, Germany; 5 Department of Pharmacology and Physiology, Saint Louis University School of Medicine, Saint Louis, MO USA

**Keywords:** Genetics, Neuroscience

## Abstract

Schaaf-Yang syndrome (SYS, OMIM #615547) is a rare neurodevelopmental disorder caused by truncating variants in the maternally imprinted *MAGEL2* gene. It is characterized by intellectual disability, autism spectrum disorder, joint contractures, and feeding difficulties. Although *MAGEL2* is deleted in most cases of Prader-Willi syndrome (PWS, OMIM #176270), SYS presents with more severe symptoms, suggesting pathogenic effects of truncated MAGEL2 beyond a mere loss of function. This study expands the behavioral characterization of a novel rat model (“*Magel2*^*Pmut*^ rats”) which carries a truncating mutation on the paternal allele of *Magel2*, offering greater construct validity for SYS than previous animal models with *Magel2* deletion. While an initial study provided first insights, key domains within the behavioral phenotype of the model remained unexplored. Our comprehensive behavioral analysis, including home-cage monitoring, ultrasonic vocalization analysis, and precise gait assessment, identified several phenotypic alterations potentially relevant to the study of SYS. *Magel2*^*Pmut*^ rats exhibited abnormal feeding behavior, changes in early social communication, alterations in gait, aberrant behavior in the elevated plus maze, and delayed decision-making. Additionally, we confirmed that *Magel2*^*Pmut*^ rats show phenotypes of abnormal social interaction. These results may reflect core symptoms seen in SYS, underscoring the value of this model for preclinical research on pathophysiology and therapeutics.

**Supplementary Information:**

The online version contains supplementary material available at 10.1038/s41598-025-20958-y.

## Introduction

The maternally imprinted gene *MAGEL2*, located on chromosome 15, is known to be critically involved in neurogenesis and various brain functions^1,2^. Its expression is elevated during pivotal stages of neurodevelopment and shows spatial enrichment in the hypothalamus^1,3,4^, a brain region that modulates a wide range of physiological processes and behaviors in humans and rodents^5^. MAGEL2 has been associated with the regulation of key neurophysiological processes, such as the biogenesis of secretory granules and neuropeptides, neurite outgrowth, and RNA metabolism^1^.

While the maternally inherited allele of *MAGEL2* is epigenetically silenced, deletions or pathogenic variants affecting the active paternal copy are of particular relevance as they are involved in the etiology of a group of neurodevelopmental disorders^6,7^. *MAGEL2* is mostly known as a protein-coding gene within the chromosomal region that is deleted in most individuals with Prader-Willi syndrome (PWS)^8^. PWS shows an estimated prevalence of 1:10,000–1:30,000 and is characterized by muscular hypotonia and failure to thrive in infancy, transitioning into food-seeking and overeating behavior as the individual ages^6,9^. Further characteristics include short stature, developmental delay, cognitive disabilities, and a behavioral profile marked by temper tantrums and outbursts^6,9^.

Truncating variants in the paternal copy of *MAGEL2*, on the other hand, cause Schaaf-Yang syndrome (SYS), a rare neurodevelopmental disorder diagnosed in over 250 individuals to date^10,11^. Although SYS shares clinical features with PWS, such as developmental delay, growth retardation, and feeding difficulties, it is distinguished by a higher prevalence of autism spectrum disorder (ASD) (approximately 75–85%) as well as joint contractures and, notably, an overall more severe phenotype^1^.

Importantly, *MAGEL2* is a single-exon gene, and variants resulting in a premature stop codon are predicted not to lead to nonsense-mediated mRNA decay, but to result in a truncated protein product^12^. MAGEL2 truncations may exert pathogenic effects beyond a mere loss of function, given that the absence of MAGEL2 in PWS patients or other individuals with smaller deletions that encompass *MAGEL2* is associated with a milder clinical presentation^1,13,14^. In fact, recent studies support the hypothesis of a neomorphic effect of truncated MAGEL2, showing that these protein variants are mislocalized predominantly to the nucleus, in contrast to the primarily cytoplasmic localization of wild-type MAGEL2^15,16^.

Behavioral alterations are known to have a great impact on disease burden as perceived by caregivers^17^. However, available therapies for SYS are limited to symptomatic treatments, which insufficiently ease the severity of the phenotype, leaving families dissatisfied^1,17^. This underscores the need to perform preclinical research to investigate SYS pathophysiology and potential treatments.

Previous mouse models have focused on complete *Magel2* deletions and thus lack construct validity for SYS, as they fail to account for the potential effects of a truncated protein^1,18^. To address this limitation, a novel rat model for SYS has been generated, carrying a truncating *Magel2* mutation (“*Magel2*^*Pmut*^ rats”)^19^. While this model offers considerable advantages over previous models—especially due to its higher construct validity for SYS and the more complex social behavior in rats compared to mice^20^—only one study has investigated it to date^19^. Initial findings of behavioral alterations in *Magel2*^*Pmut*^ rats have been reported in this first study, including changes in dyadic social interaction, perseverative-like behavior, and locomotor activity^19^. However, this work is limited by a restricted behavioral repertoire, the absence of assessments prior to weaning, and insufficient utilization of modern behavioral assessment systems^19^, which increasingly complement conventional behavioral tests and are essential for detecting subtle phenotypic differences^21,22^.

Several key domains directly relevant to the SYS phenotype, such as social communication, feeding behavior, spatial working memory, and gait, remain unexplored. Further validation of the model’s face validity, along with the identification of behavioral outcomes relevant for therapeutic intervention, is essential before it can be reliably used in preclinical treatment studies.

Here, we thoroughly characterize the *Magel2*^*Pmut*^ rat model to lay the foundation for future studies on the pathomechanistic effects of MAGEL2 truncation and therapeutic interventions. In order to detect even subtle behavioral phenotypes, we employed advanced behavioral assessment systems, such as LABORAS, a non-invasive system for monitoring rodent behavior in the home cage, ultrasonic vocalization (USV) measurements, and CatWalk XT, a precise tool for gait analysis.

## Results

**Fig. 1:**
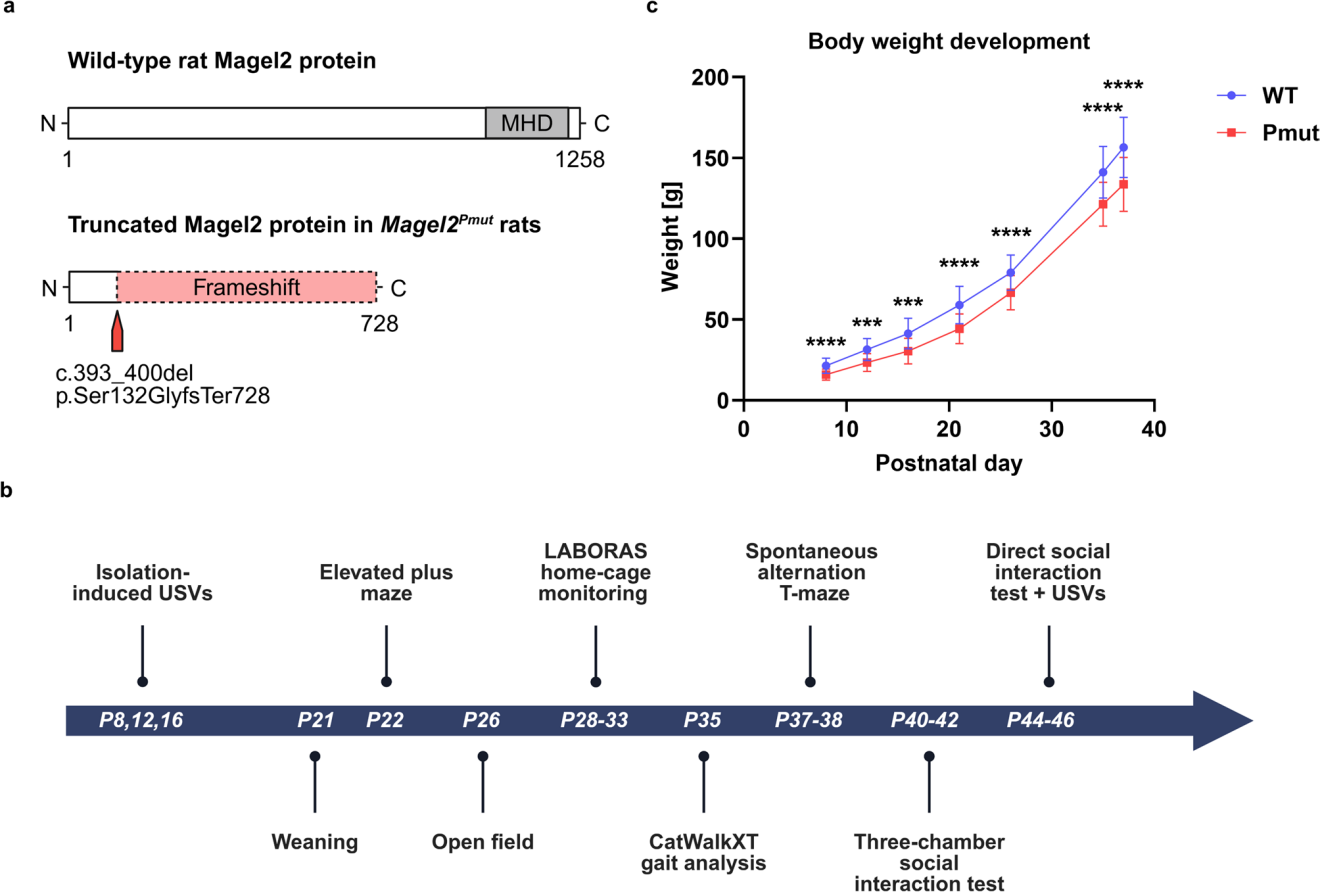
Overview of the study design and body weight development in *Magel2*^*Pmut*^ rats compared to wild-type littermates. (**a**) Illustration of the Magel2 protein in wild-type rats and *Magel2*^*Pmut*^ rats. The MAGE homology domain (MHD) in the wild-type protein, as well as the mutation site and resulting frameshift in the truncated Magel2 protein, are annotated. (**b**) Timeline showing the behavioral tests performed in *Magel2*^*Pmut*^ rats and wild-type littermates at the respective postnatal days. (**c**) *Magel2*^*Pmut*^ rats have reduced body weight compared to wild-type littermates at P8, P12, P16, P21, P26, P35, and P37. Sample sizes were *n* = 23–40 per genotype, varied slightly across time points, and are detailed in Supplementary Table S1. Data are presented as mean ± SD. Graphs show both sexes combined per genotype. Sex-specific body weight curves are provided in Supplementary Fig. S1. Although significant sex effects were detected, the absence of significant genotype-by-sex interaction indicates consistent genotype effects across sexes. ***P < 0.001, ****P < 0.0001; two-way ANOVA using genotype and sex as factors, with genotype main effects illustrated at each time point. Statistical details are provided in Supplementary Tables S1, S2.

According to the initial characterization^19^, the SYS rat model expresses a truncated Magel2 protein, as illustrated in Fig. 1a. The causative 8-base pair deletion in *Magel2* was originally annotated as c.735_742^19^, but is corrected here to c.393_400 based on Sanger sequencing and consultation with the authors of the original publication (details are provided in the methods section). Of note, we revised the previously published nomenclature *Magel2*^*m+/p−*^^19^ to *Magel2*^*Pmut*^ (indicated as Pmut; wild-type littermates indicated as WT) to better reflect the actual genetic condition and to avoid confusion with a complete knockout of the paternal allele. As *Magel2* is subject to genomic imprinting, with the maternal allele silenced and expression occurring from the paternally inherited allele^19^, *Magel2*^*Pmut*^ adequately denotes a mutation on the expressed paternal allele. This is more precise than the previous nomenclature, *Magel2*^*m+/p−*^ (maternal+/paternal-), which misleadingly suggests the paternally inherited allele is absent. To further investigate the effects of a truncating *Magel2* mutation in this animal model, we conducted a comprehensive battery of eight behavioral tests, focusing on several previously unstudied domains, including social communication, home-cage monitoring, and precise locomotion assessment (Fig. 1b).

### *Magel2*^*Pmut*^ rats display impaired weight development

Body weight of *Magel2*^*Pmut*^ and wild-type pups was monitored and compared between genotypes at different developmental stages during the first six weeks of life (P8, P12, P16, P21, P26, P35, and P37; P indicates postnatal day). *Magel2*^*Pmut*^ rats consistently showed a reduced body weight compared to wild-type littermates (Fig. 1c).

### *Magel2*^*Pmut*^ rats exhibit alterations in early social communication, which are more pronounced in male animals

Rats are known to emit a specific type of ultrasonic vocalization (USV) when separated from their mother and litter at pup age^23^. Characteristics of these 40-kHz USVs are frequently used as behavioral outcomes to measure early alterations in communication and autism-like behavior^23–25^. USVs were measured during a 5-minute isolation from the pups’ mother and littermates at P8, P12, and P16.

**Fig. 2:**
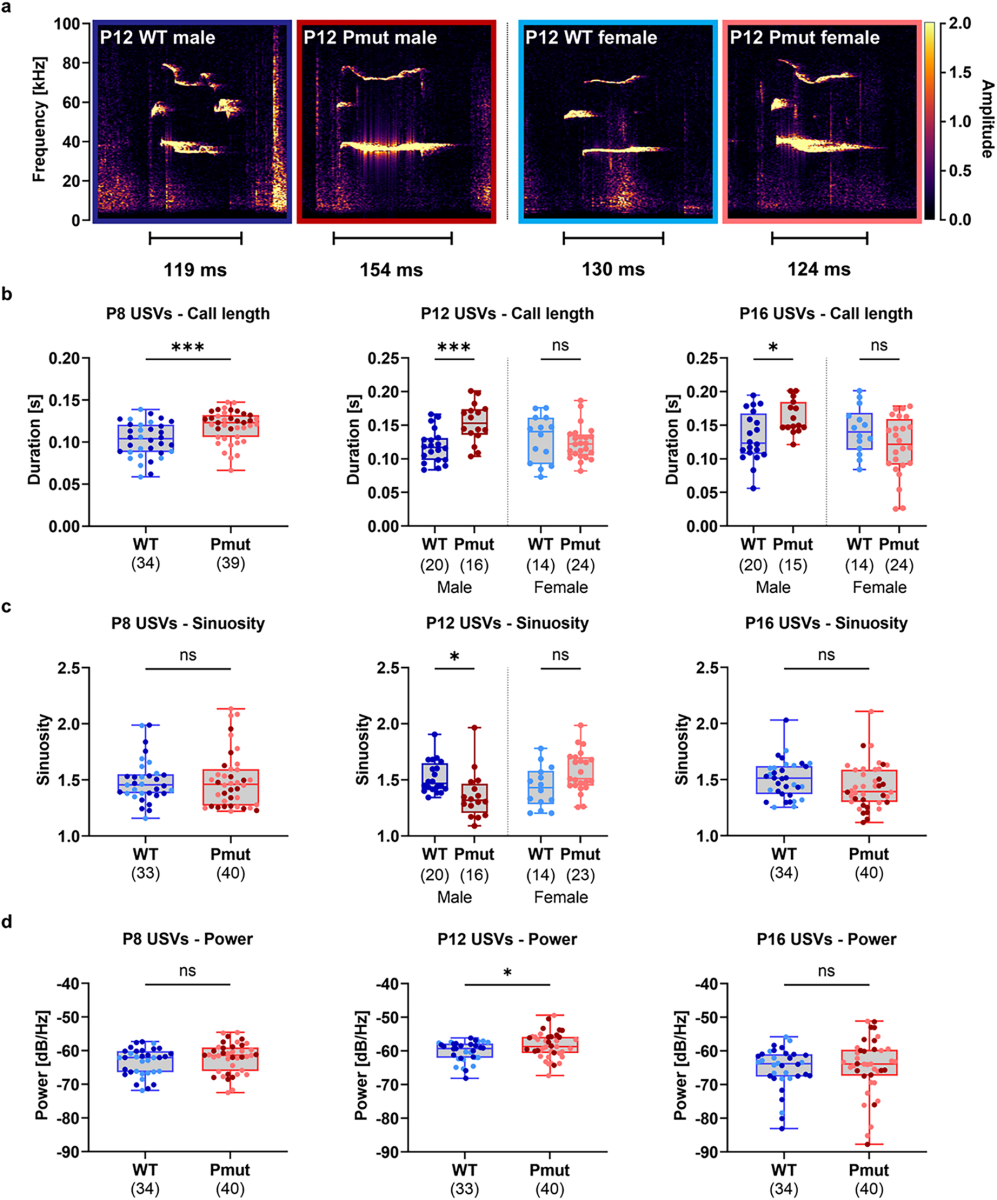
Call characteristics of isolation-induced ultrasonic vocalizations (USVs) in *Magel2*^*Pmut*^ rats compared to wild-type littermates. (**a**) Examples of calls representing the mean call duration at P12 for each genotype and sex combination. (**b**) *Magel2*^*Pmut*^ rats exhibited significantly increased call duration across both sexes at P8 and exclusively in males at P12 and P16. (**c**) Call complexity, as measured by sinuosity, was significantly reduced exclusively in male *Magel2*^*Pmut*^ rats at P12. (**d**) Call power was significantly increased in *Magel2*^*Pmut*^ rats across both sexes, exclusively at P12. Call features represent averages across all calls of each animal. Sample sizes after exclusion of outliers based on the ROUT method (Q = 1%) are indicated below the graphs. Data points for wild-type animals are shown in blue, and for *Magel2*^*Pmut*^ animals in red. Within each genotype, darker shades represent males, and lighter shades represent females. ***P < 0.001, ****P < 0.0001; ns, not significant; two-way ANOVA with genotype and sex as factors, followed by *post hoc* analyses within each sex when a significant genotype-by-sex interaction was detected. Statistical details are provided in Supplementary Tables S3–S5.

Significant differences in call length were observed at all time points (Fig. 2b). At P8, *Magel2*^*Pmut*^ animals emitted significantly longer calls compared to wild-type littermates across both sexes (Fig. 2b). However, at P12 and P16, significant genotype-by-sex interactions (Supplementary Table S4) followed by *post hoc* tests revealed that only *Magel2*^*Pmut*^ males showed significantly increased call length, while no effect was observable in females (Fig. 2b).

No significant genotype differences were observed for the number of calls or for principal, minimum, and maximum frequency (Supplementary Fig. S2). Although significant genotype-by-sex interactions were detected for some of these parameters at specific time points (Supplementary Table S4), *post hoc* tests within each sex did not reveal any significant changes (Supplementary Fig. S2).

We further examined the complexity of calls, which is frequently measured by the parameter of sinuosity^26,27^. Simple, flat, and unmodulated USVs have a sinuosity near 1, while complex calls display higher values^26^. No alterations regarding average sinuosity were observed at P8 or P16. However, a significant genotype-by-sex interaction (Supplementary Table S3) followed by *post hoc* analyses showed that male *Magel2*^*Pmut*^ rats emitted significantly less complex calls at P12 compared to their male wild-type littermates, while no significant difference was observed in females (Fig. 2c). Analysis of the average power of calls revealed a significant increase in *Magel2*^*Pmut*^ rats exclusively at P12 (Fig. 2d), while tonality was also elevated at P12 and P16 (Supplementary Fig. S2). As tonality reflects the signal-to-noise ratio of calls^26,28^, this parameter is reported here as a complementary measure to call power.

### *Magel2*^*Pmut*^ rats exhibit altered behavior in the elevated plus maze, but not in the open field test

**Fig. 3:**
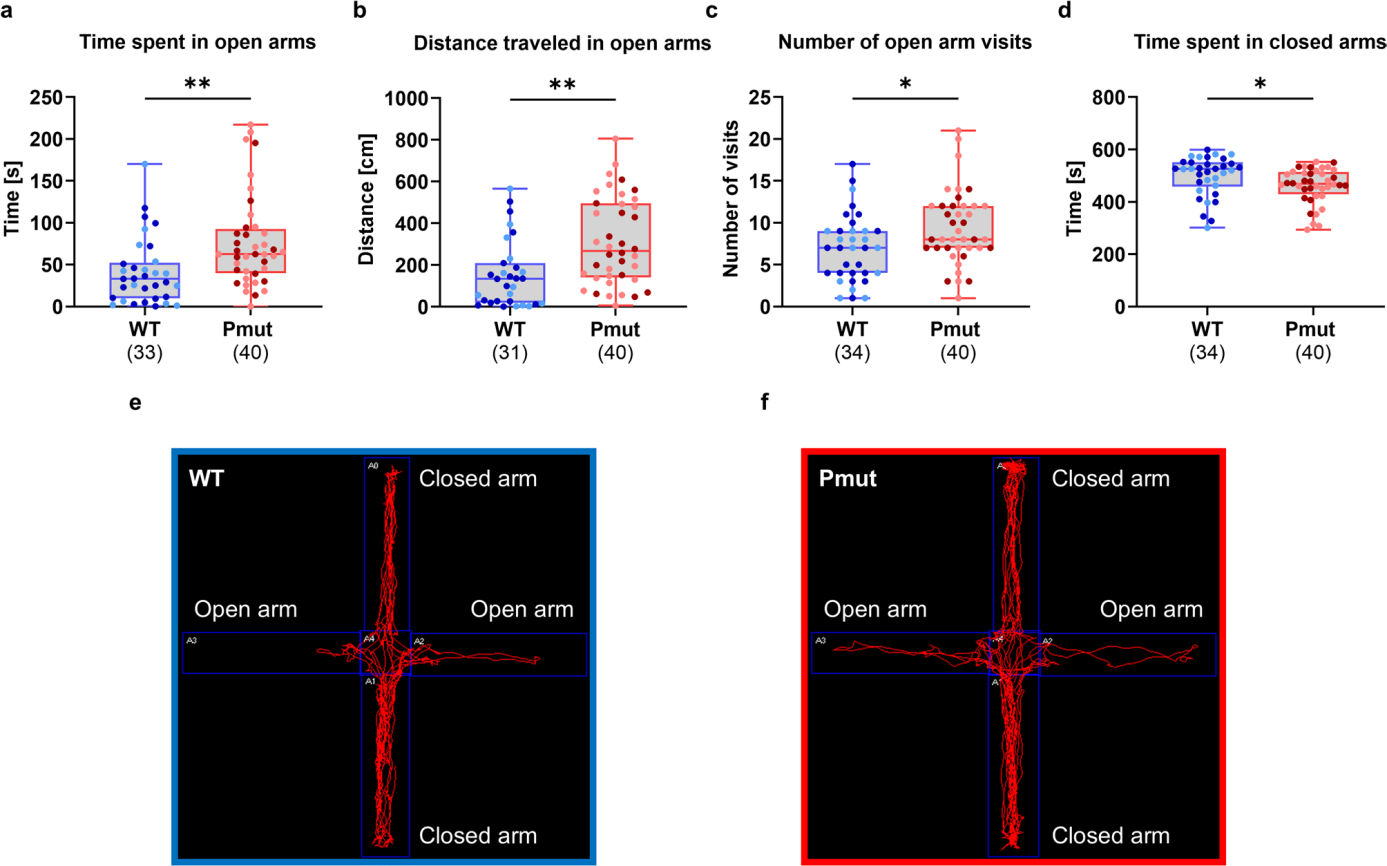
Behavioral outcomes in the elevated plus maze test in *Magel2*^*Pmut*^ rats compared to wild-type littermates. (**a**) *Magel2*^*Pmut*^ rats spent significantly more time in the open arms of the elevated plus maze compared to their wild-type littermates. (**b**) *Magel2*^*Pmut*^ rats traveled a significantly greater distance in the open arms. (**c**) A significantly higher number of visits to the open arms was observed in *Magel2*^*Pmut*^ rats. (**d**) *Magel2*^*Pmut*^ rats spent less time in the closed arms of the maze. Sample sizes after exclusion of outliers based on the ROUT method (Q = 1%) are indicated below the graphs. Data points for wild-type animals are shown in blue, and for *Magel2*^*Pmut*^ animals in red. Within each genotype, darker shades represent males, and lighter shades represent females. *P < 0.05, **P < 0.01; two-way ANOVA with genotype and sex as factors. Statistical details are provided in Supplementary Tables S6, S7. (**e**,** f**) Representative examples of track maps in the elevated plus maze for wild-type and *Magel2*^*Pmut*^ animals; since no significant sex differences or genotype-by-sex interactions were observed for any parameters, examples are not presented separately by sex.

The elevated plus maze and open field tests were used to evaluate genotype differences in behavioral domains, including anxiety-like behavior and exploratory activity. *Magel2*^*Pmut*^ rats spent significantly more time in the open arms of the elevated plus maze and traveled a greater distance within them compared to wild-type littermates (Fig. 3a, b). Additionally, they entered the open arms more frequently and spent significantly less time in the closed arms (Fig. 3c, d). Representative examples of track maps of both genotypes in the elevated plus maze can be found in Fig. 3e and f. In contrast, no significant effects were observed for any parameters in the open field test (Supplementary Fig. S3).

### *Magel2*^*Pmut*^ rats display altered feeding behavior at juvenile age, as revealed by home-cage monitoring

**Fig. 4:**
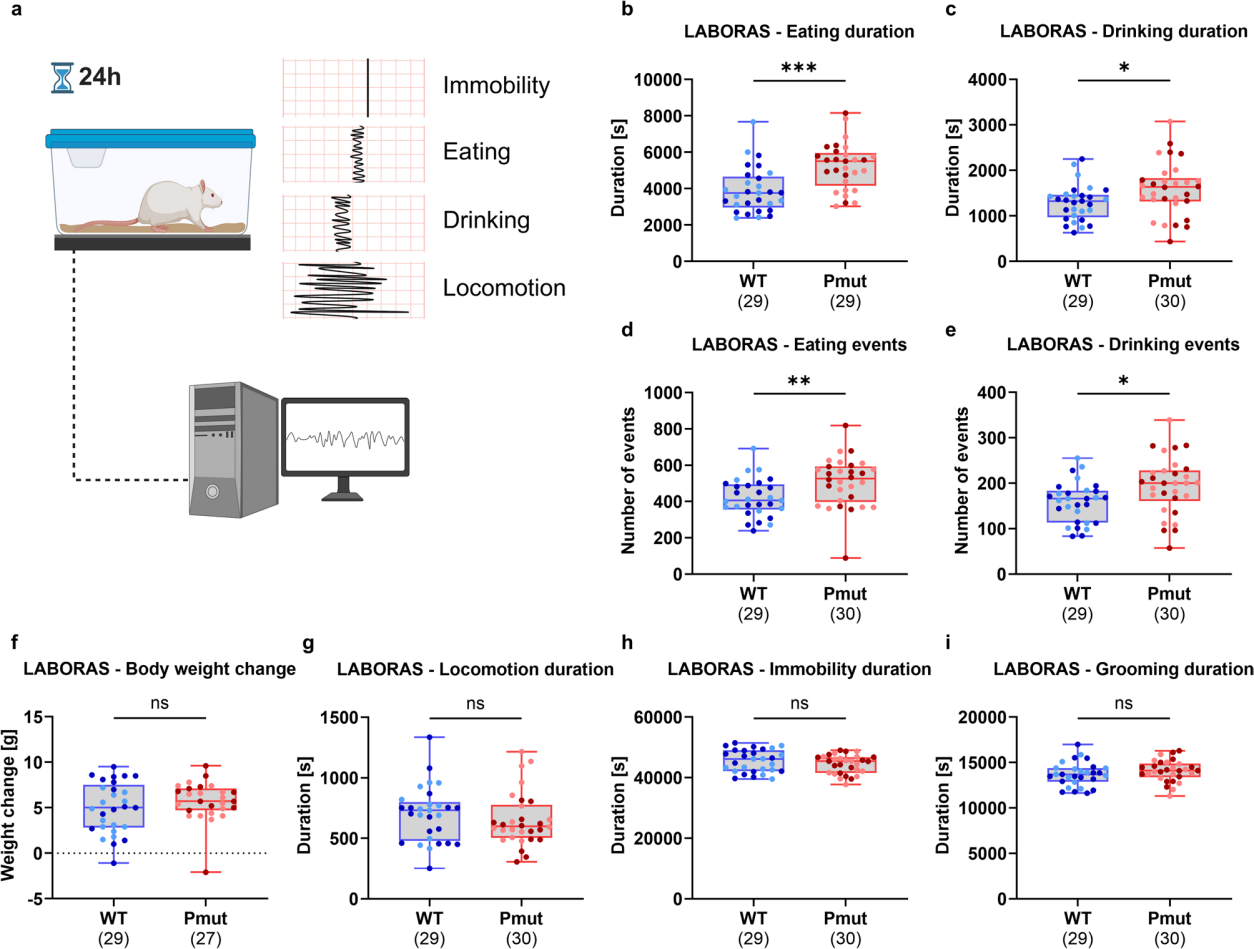
Home-cage monitoring using the LABORAS system in *Magel2*^*Pmut*^ rats compared to wild-type littermates. (**a**) Illustration of the experimental setup and the basic operating principle of the LABORAS home-cage monitoring system. (**b**,** c**) *Magel2*^*Pmut*^ rats spent significantly more time eating and drinking compared to wild-type littermates. (**d**,** e**) *Magel2*^*Pmut*^ rats exhibited a significantly increased number of eating and drinking events. (**f**) Change in body weight during the 24-hour home-cage monitoring period was comparable between wild-type and *Magel2*^*Pmut*^ rats. (**g–i**) Locomotion, immobility, and grooming behavior did not significantly differ between genotypes. Sample sizes after exclusion of outliers based on the ROUT method (Q = 1%) are indicated below the graphs. Data points for wild-type animals are shown in blue, and for *Magel2*^*Pmut*^ animals in red. Within each genotype, darker shades represent males, and lighter shades represent females. *P < 0.05, **P < 0.01, ***P < 0.001; ns, not significant; two-way ANOVA with genotype and sex as factors. Statistical details are provided in Supplementary Tables S10, S11.

The LABORAS automated home-cage monitoring system was used to comprehensively characterize the undisturbed behavior of the rats for an observation period of 24 h. Animals were tested at juvenile age between P28 and P33. This system tracks vibrations and force signals induced by different movements using a carbon fiber measurement plate and reliably identifies a vast range of behaviors by their unique signal characteristics (Fig. 4a)^21^. Despite their reduced weight during the first weeks of life (Fig. 1c), *Magel2*^*Pmut*^ animals ate and drank significantly longer compared to wild-type littermates (Fig. 4b, c). Additionally, the number of eating and drinking events was also significantly increased in mutant animals (Fig. 4d, e). Despite those differences, the change in body weight during the 24-hour observation period was comparable between genotypes (Fig. 4f). Other behaviors, including locomotion, immobility, and grooming, did not differ significantly between *Magel2*^*Pmut*^ animals and wild-type littermates regarding either duration or number of events (Fig. 4g–i, Supplementary Fig. S4). Also, the total distance traveled was comparable between genotypes (Supplementary Fig. S4).

### Juvenile *Magel2*^*Pmut*^ rats exhibit altered gait parameters without changes to average speed

**Fig. 5:**
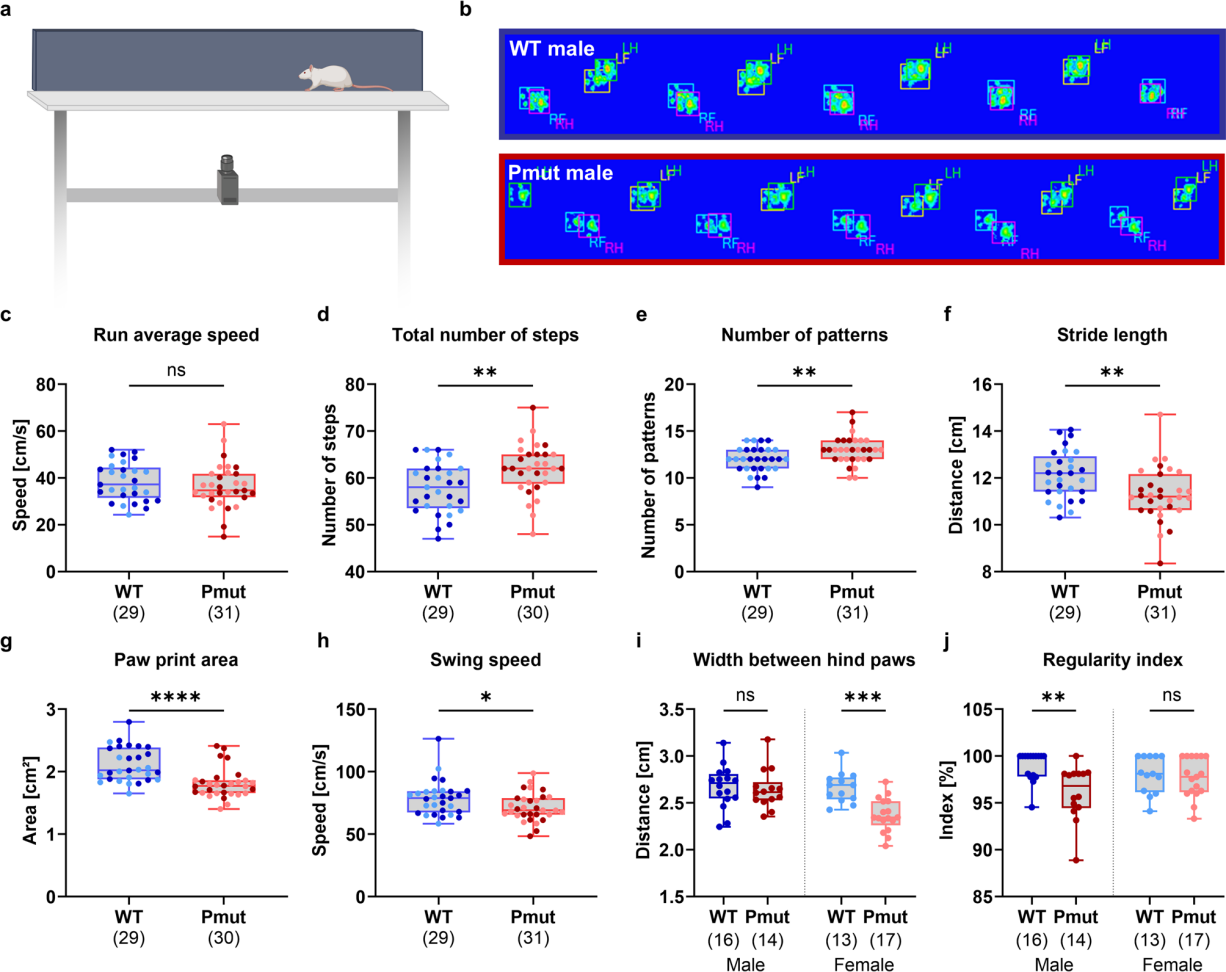
CatWalk XT gait analysis in *Magel2*^*Pmut*^ rats compared to wild-type littermates. (**a**) Schematic illustration of the CatWalk XT system setup. (**b**) Representative footfall patterns from a single run in male wild-type and *Magel2*^*Pmut*^ rats, regarding stride length, paw print area, and regularity index. (**c**) Run average speed was comparable between *Magel2*^*Pmut*^ and wild-type rats. (**d**,** e**) *Magel2*^*Pmut*^ rats exhibited a significantly increased number of steps and gait patterns. (**f–h**) Stride length, paw print area, and swing speed were significantly reduced in mutant animals. (**i**) Width between hind paws was significantly decreased in female *Magel2*^*Pmut*^ rats, but not in males. (**j**) Step regularity was significantly decreased in male *Magel2*^*Pmut*^ rats, but not in females. Sample sizes after exclusion of outliers based on the ROUT method (Q = 1%) are indicated below the graphs. Data points for wild-type animals are shown in blue, and for *Magel2*^*Pmut*^ animals in red. Within each genotype, darker shades represent males, and lighter shades represent females. *P < 0.05, **P < 0.01, ***P < 0.001, ****P < 0.0001; ns, not significant; two-way ANOVA with genotype and sex as factors, followed by *post hoc* analyses within each sex when a significant genotype-by-sex interaction was detected. Statistical details are provided in Supplementary Tables S12–S14.

An automated gait analysis system (CatWalk XT) was used to comprehensively assess motor function and gait in the SYS rat model. Animals voluntarily crossed a glass walkway, beneath which a camera was positioned to capture their footprints, as illustrated in example images, during three runs from one end to the other (Fig. 5a, b).

While *Magel2*^*Pmut*^ rats did not show significant changes in average run speed or run duration, they required a significantly greater number of steps and footfall patterns to cross the walkway compared to wild-type littermates (Fig. 5c–e, Supplementary Fig. S5). Consistent with these findings, which may reflect a general delay in physical development, *Magel2*^*Pmut*^ animals also exhibited a significantly decreased stride length, paw print area, paw print length, and paw print width, as well as reduced swing speed during their steps (Fig. 5f–h, Supplementary Fig. S5). In contrast, stand time, swing time, and cadence (the number of steps per second) were unchanged (Supplementary Fig. S5).

The width between front paws did not differ significantly between genotypes (Supplementary Fig. S5). However, a significant genotype-by-sex interaction was observed for the width between the hind paws (Supplementary Table S13), with *post hoc* analyses revealing that female but not male *Magel2*^*Pmut*^ rats exhibited a significantly narrower hind paw stance compared to the respective wild-type control group (Fig. 5i).

We additionally assessed different support parameters that describe the proportion of time animals spent supporting their weight on specific combinations of paws^22^ (Supplementary Fig. S5). These included support by a single paw, diagonal pairs of paws (e.g., left front paw and right hind paw), lateral pairs (e.g., right front paw and right hind paw), three paws, and all four paws. However, no significant genotype differences were observed (Supplementary Fig. S5).

Interestingly, we observed a change in the regularity index, which quantifies interlimb coordination performance in rodents^29^. In healthy wild-type animals with normal gait patterns, this index typically approaches 100%, while the presence of missteps disrupting the regular step sequence results in a reduced regularity index^29^. A significant genotype-by-sex interaction was found (Supplementary Table S13), and *post hoc* tests revealed a significant reduction in the regularity index only in *Magel2*^*Pmut*^ males (Fig. 5j).

### *Magel2*^*Pmut*^ rats require more time but make accurate decisions in a cognitive flexibility task

The spontaneous alternation T-maze paradigm was used to assess spatial working memory and cognitive flexibility in *Magel2*^*Pmut*^ rats and wild-type littermates. In this task, rats repeatedly choose between the left and right arms of a T-maze. Typically, they remember their previous choice and tend to select the opposite arm in the next trial, resulting in an alternation rate of approximately 75% in wild-type animals^30^.

**Fig. 6:**
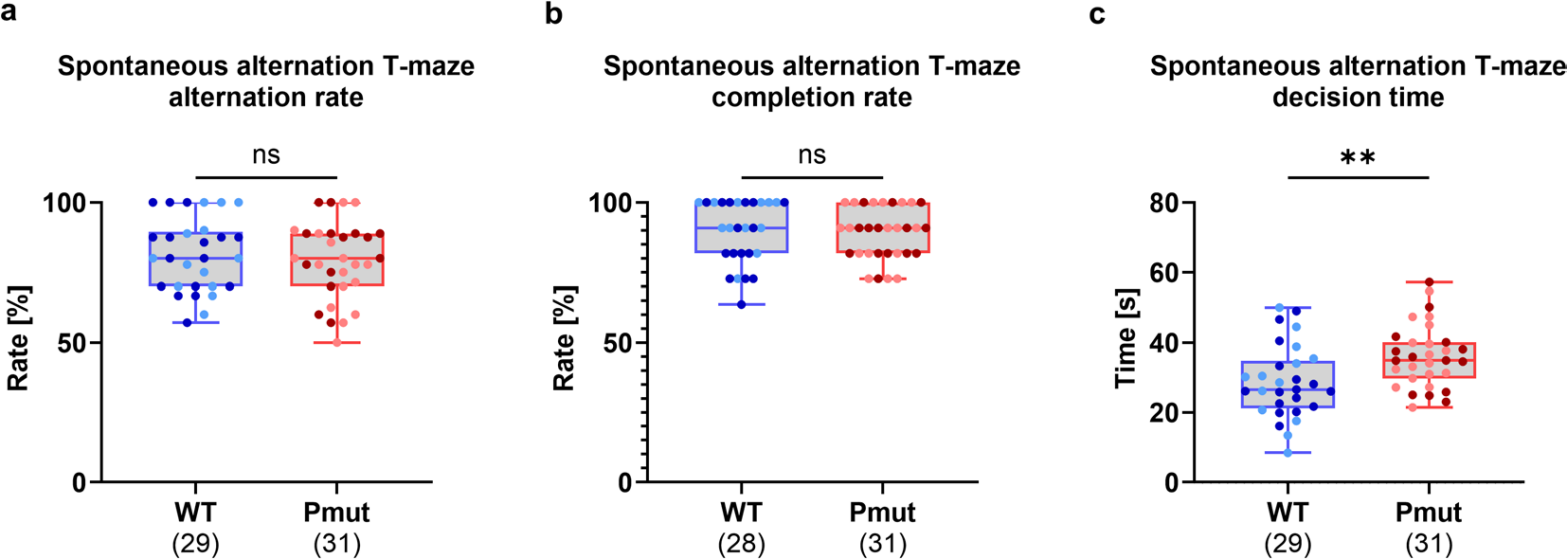
Spatial working memory in *Magel2*^*Pmut*^ rats compared to wild-type littermates assessed using the spontaneous alternation T-maze paradigm. (**a**,** b**) No significant differences in alternation or completion rates were observed between *Magel2*^*Pmut*^ rats and wild-type littermates. (**c**) The average decision time was significantly increased in *Magel2*^*Pmut*^ rats compared to wild-type littermates. Sample sizes after exclusion of outliers based on the ROUT method (Q = 1%) are indicated below the graphs. Data points for wild-type animals are shown in blue, and for *Magel2*^*Pmut*^ animals in red. Within each genotype, darker shades represent males, and lighter shades represent females. **P < 0.01; ns, not significant; two-way ANOVA with genotype and sex as factors. Statistical details are provided in Supplementary Tables S15, S16.

*Magel2*^*Pmut*^ rats and wild-type littermates both exhibited alternation rates close to this expected level, with no significant difference observed (Fig. 6a). To account for potential differences in task engagement, we normalized the alternation rate by the number of completed trials, as previously suggested^31^. Trials were terminated after 2 min if no arm was chosen, and the trial completion rate was evaluated separately as a measure of motivation. No significant change in trial completion was found between genotypes, with both groups completing approximately 90% of trials (Fig. 6b). In contrast, when analyzing the decision time, reflecting the memory retrieval and decision-making process, we found that *Magel2*^*Pmut*^ animals exhibited significantly longer decision times compared to wild-type littermates (Fig. 6c).

### *Magel2*^*Pmut*^ rats show qualitative changes in direct social interaction but no alterations in general sociability or social novelty

The three-chamber social interaction test was used to assess general sociability and the preference for social novelty in *Magel2*^*Pmut*^ rats and wild-type littermates by automatically analyzing the time spent in designated compartments of an arena containing different social stimuli (Supplementary Fig. S6). However, no significant differences were observed in any of the measured parameters (Supplementary Fig. S6).

**Fig. 7:**
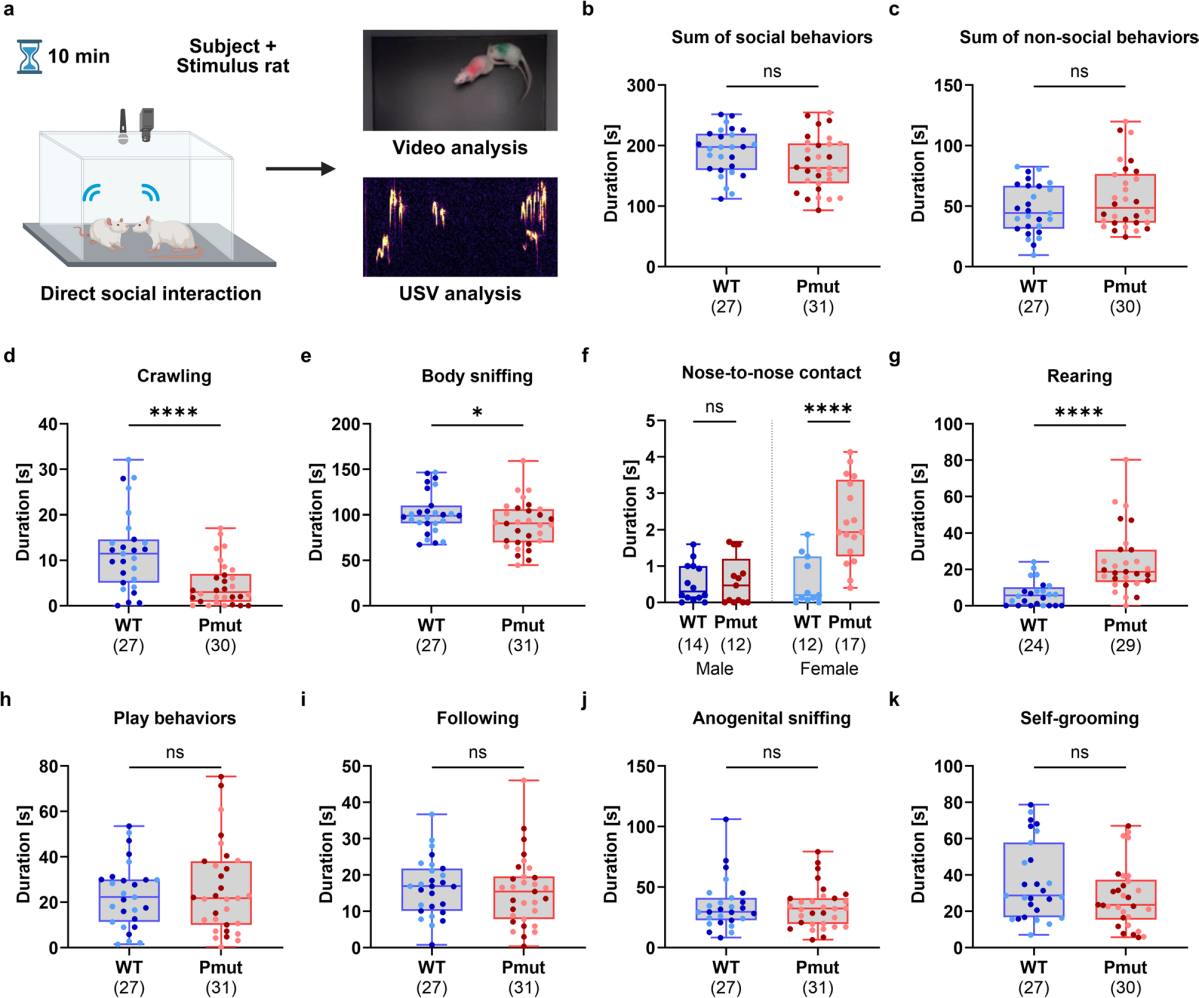
Direct social interaction test in *Magel2*^*Pmut*^ rats compared to wild-type littermates. (**a**) Schematic illustration of the experimental setup and analysis pipeline. (**b**,** c**) No significant differences were observed in the total time spent with social or non-social behaviors between *Magel2*^*Pmut*^ rats and wild-type littermates. (**d**,** e**) *Magel2*^*Pmut*^ rats spent significantly less time with crawling and body sniffing behavior, respectively. (**f**) The duration of nose-to-nose contact was significantly increased in female *Magel2*^*Pmut*^ rats, but not in males, compared to their respective wild-type controls. (**g**) Rearing duration was significantly increased in *Magel2*^*Pmut*^ rats. (**h–k**) The duration of play behaviors, following behavior, anogenital sniffing, and self-grooming did not differ significantly between genotypes. Sample sizes after exclusion of outliers based on the ROUT method (Q = 1%) are indicated below the graphs. Data points for wild-type animals are shown in blue, and for *Magel2*^*Pmut*^ animals in red. Within each genotype, darker shades represent males, and lighter shades represent females. *P < 0.05, ****P < 0.0001; ns, not significant; two-way ANOVA with genotype and sex as factors, followed by *post hoc* analyses within each sex when a significant genotype-by-sex interaction was detected. Statistical details are provided in Supplementary Tables S19–S21.

To gain deeper insights into details of social behavior and communication, we subsequently conducted an in-depth video and ultrasonic vocalization (USV) analysis based on a 10-minute social encounter between the test animals and an unfamiliar, age- and sex-matched, wild-type stimulus rat (Fig. 7a). During this encounter, the subject rat was scored for different kinds of social behaviors, such as crawling, sniffing, and play behaviors, as well as for non-social behaviors, including self-grooming and rearing. Although total time spent with social or non-social behaviors did not differ significantly between genotypes (Fig. 7b, c), we observed changes in specific behavioral subcategories. *Magel2*^*Pmut*^ rats spent significantly less time crawling over or under the stimulus rat and sniffing its body compared to wild-type littermates (Fig. 7d, e). A significant genotype-by-sex interaction was observed for nose-to-nose contact duration (Supplementary Table S20) and *post hoc* analyses revealed a significantly increased duration in female *Magel2*^*Pmut*^ animals, but not in males (Fig. 7f). Additionally, rearing, a non-social behavior often associated with exploratory behavior, was drastically increased in *Magel2*^*Pmut*^ animals compared to wild-type littermates (Fig. 7g). No significant differences were observed in the duration spent on play behaviors, following behavior, anogenital sniffing, or self-grooming (Fig. 7h–k).

To complement the video analysis, we also assessed 50-kHz USVs emitted during the encounter, which are linked to a positive emotional state^23^. Since we were unable to distinguish between the calls of the experimental and the stimulus rats, we compared wild-type-wild-type pairings with *Magel2*^*Pmut*^-wild-type pairings. Although a trend was observed towards reduced prosocial 50-kHz USVs in pairs involving *Magel2*^*Pmut*^ rats (two-way ANOVA, genotype effect *P* = 0.0586), none of the vocalization parameters analyzed showed significant genotype differences (Supplementary Fig. S7).

## Discussion

Schaaf-Yang syndrome is a severe neurodevelopmental disorder^1^. Animal models with good construct and face validity are necessary to advance our understanding of pathophysiology and to serve as tools for preclinical therapeutic intervention. The recently developed rat model of SYS holds promise in this context. However, knowledge of its phenotype and face validity remains limited, with only a single publication describing the model to date^19^. Here, we present an expanded phenotypic characterization of the *Magel2*^*Pmut*^ rat model, incorporating new and relevant behavioral assays. We also extended the assessment of social and anxiety-like behavior to confirm and refine previous findings. These comprehensive analyses reveal previously unreported phenotypic alterations in this animal model, which may be interpreted as reflecting symptoms of SYS.

The earliest behavioral deviations in children with SYS include atypical crying at birth and difficulties with emotional expression^1,32^. To investigate comparable behaviors in our animal model, rat pup ultrasonic vocalizations (USVs), frequently compared to human infant crying^33,34^, served as markers reflecting early changes in affective and social behavior^23^. *Magel2*^*Pmut*^ rats exhibited significantly prolonged, more powerful, and less complex calls at specific developmental stages compared to wild-type littermates. Some of these effects were seen consistently across both sexes, while others were observed exclusively in males. This suggests a phenotype of altered early social communication, with a more pronounced manifestation in male animals. Interestingly, prolonged call durations of isolation-induced USVs but normal call numbers were also observed in homozygous *Cntnap2* knockout pups bred from heterozygous parents, another ASD rat model^24^. The authors emphasize the use of USV structure analysis to quantify ASD-like traits, with alterations in vocal social communication being one of the core domains to assess^24^. The findings of altered USV parameters in *Magel2*^*Pmut*^ rats are therefore particularly interesting given the high prevalence of ASD in SYS, estimated at approximately 75 to 85%^11^, with communication deficits affecting the vast majority of children^32^.

The more pronounced effects we observed in males align with findings from other ASD rodent models^35,36^ and are interesting in light of the generally higher male susceptibility to autistic behavioral impairments, as observed in humans across various neurodevelopmental disorders^37^. However, it is important to note that SYS is characterized by a high penetrance of ASD, even in affected females, highlighting that these pronounced effects in males may not fully represent what is seen specifically in individuals with SYS^1^. Ongoing studies exploring sex-specific phenotypic differences in SYS will provide better context for these findings, as well as for other sex-specific differences observed in this study.

Moreover, while these changes may reflect symptoms observed in SYS and could serve as a valuable behavioral outcome in this model, future studies are necessary to determine whether these altered call characteristics have functional relevance for maternal retrieval behavior.

To assess whether *Magel2*^*Pmut*^ rats exhibit alterations in social behavior or social communication at later developmental stages, potentially mirroring further aspects of ASD, we conducted the three-chamber social interaction test (TCT), the direct social interaction (DSI) test, and recorded USVs during encounters with an unfamiliar conspecific. Our findings indicate that *Magel2*^*Pmut*^ rats do not show major deficits in sociability or social recognition relative to wild-type controls, as assessed by the TCT. This extends the first characterizations of this model, which only evaluated sociability but similarly reported no deficit^16^. However, more subtle changes in social behavior were detectable in the DSI test. *Magel2*^*Pmut*^ rats spent significantly less time engaging in certain behaviors such as crawling over or under the stimulus rat and body sniffing, while the non-social behavior of rearing was markedly increased. These alterations may represent a shift in social engagement patterns, altered social communication strategies, or an increase in environmental exploration during social interaction, each of which could contribute to the qualitative behavioral differences observed. However, a general deficit in social motivation in *Magel2*^*Pmut*^ rats appears unlikely, as we did not find alterations regarding sociability in the TCT or a difference in the overall time spent with social behaviors in the DSI test. Our results align well with the previous findings of qualitative changes in social interaction in this rat model, even though the specific manifestations are different^19^. This is likely attributable to differences in the age at which animals were tested, given that social behavior in rats is highly dynamic during this developmental stage^38^, as well as variations in experimental protocols and the specific behaviors scored.

We also observed a trend towards reduced prosocial 50-kHz USVs emitted during social encounters involving *Magel2*^*Pmut*^ rats. Given the limitations of conventional USV recording during social interaction, particularly the inability to distinguish calls from individual animals during interaction, future studies using improved methods (e.g., miniature microphones to assign a vocalization to a specific individual^39^ are needed to determine whether communication during social interaction is indeed impaired in this model.

We further found significantly increased time spent in the open arms of the elevated plus maze, along with other altered parameters, possibly indicating a reduction in anxiety-like behavior, altered risk assessment, impulsivity, or perseverative locomotor patterns in *Magel2*^*Pmut*^ rats. These findings complement the first study of this animal model, which used the elevated circle maze, a paradigm based on the same principle and similarly effective at detecting anxiety-like behavior^40^. However, the initial study reported a significant increase in time spent in the open region only in rats homozygous for the truncating *Magel2* mutation (which were not evaluated in the present study, as they less accurately recapitulate the genetic background of SYS), whereas no significant changes were observed in heterozygous *Magel2*^*Pmut*^ rats^19^. Although heightened anxiety is generally observed in individuals with SYS, they paradoxically also exhibit a reduced awareness of danger^1,32^, a characteristic that may be reflected in our findings in the SYS model, given the established interpretation that rodents typically perceive open regions as potentially dangerous^41^. However, especially considering that repetitive behaviors (as a core symptom of ASD) and impulsivity are also prominent features of SYS^1^, alternative explanations for the observed behavior—such as perseverative locomotor patterns or heightened impulsive tendencies—should also be taken into account.

Feeding difficulties during infancy affect nearly all individuals with SYS, often necessitating assisted feeding techniques^11,17,42^. Early life home-cage monitoring of *Magel2*^*Pmut*^ rats revealed a significantly increased time spent eating and drinking, as well as more eating and drinking events compared to wild-type littermates. Despite this, 24-hour body weight gain remained unchanged, and mutant rats exhibited markedly reduced body weight during this developmental period, as observed both in our study and by Reznik et al.^19^. Several hypotheses could explain why mutant animals spend more time eating and drinking to achieve similar weight gain as wild-type littermates. We consider ineffective oral intake, consistent with the clinical presentation of SYS patients, to be a plausible explanation. However, other mechanisms, such as increased energy expenditure, impaired nutrient absorption, or metabolic inefficiencies, cannot be ruled out. In the absence of direct measurements of energy intake and expenditure, this interpretation should therefore be regarded as a hypothesis. Future studies employing metabolic cages to precisely quantify food and water intake, as well as energy balance, will be critical for its validation and for supporting preclinical research on this key symptom of SYS.

Additionally, muscular hypotonia and joint contractures are almost universally present in SYS, while short stature and scoliosis are seen in about half of cases^11^. To investigate whether the SYS rat model exhibits alterations related to these patient phenotypes, we employed a detailed gait analysis that can detect even subtle changes in motor function^43^. This approach expands upon the previous work, which to date has relied exclusively on the open field test to assess gross locomotor activity^19^. *Magel2*^*Pmut*^ rats exhibited consistent alterations in spatial gait parameters compared to wild-type littermates, including significantly reduced stride length, paw print area, as well as an increased number of steps needed to cross the walkway. Additionally, they showed a significantly reduced swing speed of the paws. Given the concurrent reduction in body weight observed in mutant animals, we interpret these changes primarily as a consequence of smaller body size, mirroring the short stature observed in individuals with SYS, although neuromuscular factors or joint limitations could also contribute to the changes. To further explore potential functional deficits beyond size-related differences, we analyzed the regularity index, a well-established parameter for assessing overall interlimb coordination^29,43^. Notably, male *Magel2*^*Pmut*^ rats displayed a significantly reduced regularity index, which may reflect subtle coordination deficits potentially mirroring aspects of SYS including hypotonia and joint contractures. However, we note that other outcomes such as run speed, cadence (number of steps per second), stand and swing time, as well as support metrics, were unchanged. This indicates that the observed phenotype may be relatively mild and that mutant rats are able to compensate for coordination and stature-related deficits.

We did not normalize gait parameters for body weight, as short stature is a common clinical feature of SYS, and we regard reduced body size, which is reflected in several parameters, as an integral part of the phenotype of this model. It therefore should be captured rather than adjusted for, particularly given its role as a treatment target. For instance, those phenotypes could serve as valuable outcomes in the study of recombinant growth hormone therapy, which has been proposed as a treatment addressing short stature in SYS^1,44^.

Previous work on this rat model has reported no significant deficits in the novel object recognition or fear conditioning paradigms, suggesting broadly preserved recognition memory and associative learning^19^. Our findings extend this behavioral characterization by showing that mutant animals exhibited significantly prolonged decision times in the spontaneous alternation T-maze task, while alternation rates remained comparable to wild-type littermates. Trial completion rates were also unaffected, and gait alterations, observed in the model, did not result in functional impairments in parameters such as average gait speed or total distance traveled during 24 h of home-cage monitoring. Therefore, substantial motivational, attentional, or motor function deficits appear unlikely to account for the observed delay in decision-making. Although a general impairment in memory retrieval does not appear to be present in *Magel2*^*Pmut*^ rats in the spontaneous alternation task, as indicated by preserved alternation rates, the observed prolongation of decision times may, to some extent, indicate slowed cognitive processing speed and, consequently, altered cognitive flexibility. While this alteration is subtle, it may reflect aspects of the SYS phenotype, which always includes intellectual disability ranging from mild to profound cases^11,12^.

Interestingly, some behavioral domains assessed in this study revealed sex-dependent differences in the phenotype of *Magel2*^*Pmut*^ rats. However, a detailed clinical contextualization of these changes is currently not possible, as to date, studies on the clinical phenotype of SYS itself have not performed sex-stratified analyses, with the sole exception of hypogonadism prevalence as reported by McCarthy et al.^12^. It is, however, noteworthy that in *Magel2* knockout mice, a female-specific social phenotype has also been reported, which bears some resemblance to the increased duration of nose-to-nose contact observed in the present study. Specifically, female *Magel2* knockout mice displayed longer baseline interaction times in the partition test^45^. Although sex-associated methylation of the *MAGEL2* promoter has been suggested^46^, potential sex-related differences in underlying biological mechanisms in SYS or corresponding model systems remain largely unexplored. RNA-seq analyses of male and female neurons, both in the rat model and in patient-derived cell lines, would be particularly valuable to systematically address this gap.

Limitations of this study include general challenges inherent to using rodent models to study neurobehavioral disorders and specific methodological considerations. The first category revolves around the fact that determining the extent to which findings in rodent models correspond to human disorder, and vice versa, is difficult^47^. For instance, it remains unclear whether studying rodent social behavior in our established paradigms can be adequately compared to the nuanced characteristics of human social interaction^47^. Additionally, factors such as high intersubject variability in rodent behavioral measures^47,48^ further complicate efforts to model the full spectrum of human symptoms.

A more specific limitation of this study concerns the genetic variant in the *Magel2*^*Pmut*^ rat model. Although this variant results in a truncated protein similar in terms of size and domain loss to the expected truncated protein in individuals with the most common SYS variant (c.1996dupC)^19^, important differences remain. These include the earlier position of the variant in the rat model, the longer frameshift, and the different amino acid sequence of the resulting protein product. Additionally, given the broad phenotypic spectrum of SYS and the variety of causative variants^1^, a single animal model cannot capture the full complexity of the disease. Investigating other truncating variants in rodent models could help complement clinical studies on disease heterogeneity and genotype-phenotype correlations.

Some phenotypical alterations observed in the SYS rat model, including aberrant behavior in the elevated plus maze, changes in isolation-induced USVs, and growth retardation, are consistent with those reported in *Magel2* knockout mouse models^45,49,50^. However, a direct comparison to assess whether the truncating mutation in *Magel2*^*Pmut*^ rats results in a more severe phenotype compared to models with complete *Magel2* deletions, paralleling findings in humans, remains challenging. Several factors contribute to this limitation: (1) the models were developed in different species; (2) the rat model has not been studied as extensively as the mouse model; and (3) even within two different *Magel2* knockout mouse models, relevant phenotypical differences exist^18^.

Nevertheless, given the considerably higher construct validity of *Magel2*^*Pmut*^ rats for SYS, we consider this model an important addition and advocate for its further investigation to study the role of truncated MAGEL2 in disease pathophysiology and to test therapeutic approaches for SYS.

In summary, we provide novel insights into the phenotype of a rat model of SYS. Specifically, the presented alterations in early social communication, feeding behavior, social interaction, and gait may reflect symptoms observed in affected individuals, thereby supporting the face validity of this model. Currently discussed treatment strategies for SYS include hormone replacement therapies and administration of oxytocin during a critical developmental period^1,51^. Additionally, antisense oligonucleotide (ASO) treatment aimed at reducing truncated MAGEL2 has been proposed and could be a promising approach, especially combined with therapeutic strategies targeting reactivation of the maternal copy of *MAGEL2*^1^. It will be crucial to further investigate the efficacy and potential side effects of these therapies in a model that closely reflects the genetic background of the disease. The *Magel2*^*Pmut*^ rat model appears promising in this regard and warrants further validation.

## Methods

### Animals

All animal experiments were approved by the Animal Ethics Committee of the Regierungspräsidium Karlsruhe, Germany (G173/21, G175/21, G176/21, and G177/21) and were performed in accordance with the relevant guidelines and regulations. All experiments were conducted in accordance with the ARRIVE guidelines. Rats were housed in a 12-h dark-light cycle at 22 +/- 2 °C and had access to food and water *ad libitum*. All animals had a Sprague-Dawley background. Breeding was performed by mating wild-type females to *Magel2*^*Pmut*^ males, resulting in approximately equal numbers of wild-type and *Magel2*^*Pmut*^ offspring. A detailed description of the generation of the *Magel2* mutation can be found in the first publication describing this model^19^. At P7, animals received a paw tattoo for identification purposes. At P21, ear punch biopsies were taken, and animals were weaned and assigned to three to five rats per cage. All cages contained a small tube as enrichment. After completion of behavioral studies, animals were euthanized with an overdose of isoflurane following the American Veterinary Medical Association (AVMA) Guidelines for the Euthanasia of Animals.

Age-matched wild-type Sprague-Dawley rats that served as stimulus rats for the three-chamber test and direct social interaction test were separately ordered from Janvier Laboratories. Stimulus animals were habituated to the testing facility for at least seven days before the respective paradigm.

All experimental procedures were performed at the Interdisciplinary Neurobehavioral Core (INBC) of Heidelberg University, Germany. Testing took place during the light cycle. If not stated otherwise, experiments were conducted at dim light conditions (< 30 lx). The same experimenter conducted all behavioral assessments and remained blinded to the genotypes until the completion of all testing. After each assessment, the respective apparatus was cleaned with 70% ethanol before the next animal was tested.

Genotyping of animals was performed by PCR as previously described^19^. The position of the 8-base pair deletion in *Magel2*^*Pmut*^ rats was confirmed by Sanger sequencing of both genomic DNA (gDNA) and complementary DNA (cDNA). gDNA sequencing of *Magel2*^*Pmut*^ rats was performed to confirm heterozygosity and identify the base pair at which the deletion begins. This was determined based on the appearance of overlapping peaks in the chromatogram, indicating the point at which the wild-type and mutant allele sequences no longer aligned. cDNA sequencing was performed to confirm the deletion resided on the expressed paternal allele, to validate that there was no evidence of “leaky” expression from the transcriptionally silent wild-type maternal allele, and to verify the precise length of the deletion. gDNA was extracted from tail biopsies using phenol-chloroform. To generate cDNA, total RNA was extracted from brain tissue with Trizol, treated with DNase (Thermo Scientific, Waltham, MA, USA), and reverse-transcribed into cDNA using the ProtoScript II First Strand cDNA Synthesis Kit (New England Biolabs, Ipswich, MA, USA) following the manufacturer’s protocol. For both gDNA and cDNA, PCR amplification was performed using the following primers: forward 5′-AAAAAGCGTAGCAACCGGAG-3′ and reverse 5′-GGAGGGAGAGGCTGGACC-3′. PCR products were purified using the QIAquick PCR Purification Kit (Qiagen, Hilden, Germany). Sanger sequencing was performed by Microsynth Seqlab GmbH (Göttingen, Germany), and the results were analyzed with SnapGene software (GSL Biotech LLC, Chicago, IL, USA). The position of the mutation site identified as c.393_400 corresponds to the translated truncated protein described by Reznik et al., which was confirmed to be present in the rat model by mass spectrometry^19^. The error in the original publication was solely in the annotation of the nucleotide-level change (mistakenly reported as c.735_742^19^, not the amino acid-level change.

### Isolation-induced ultrasonic vocalizations

We measured ultrasonic vocalizations (USVs) at P8, 12, and 16 using an established paradigm in rodents^23–25,52^.

Rat pups were isolated individually from their mother and placed in a glass container (21 cm x 9 cm x 21 cm) that was lined with paper towels at its bottom to minimize background noises during the recordings. The glass container was then cautiously placed in a sound-attenuated chamber (42 cm x 42 cm x 42 cm) with a closed top. 30 cm above the ground, an UltraSoundGate condenser microphone (CM16/CMPAc) was installed that was connected to a computer through an Avisoft UltraSoundGate USG416H audio device. 5 min were recorded per animal using Avisoft-RECORDER software (Avisoft Bioacoustics, Glienicke, Germany) at a 250-kHz sampling rate. All audio signals were stored as WAV files.

Recordings were automatically analyzed using DeepSqueak (Version 3.1.0) in MATLAB version R2024a^28^. DeepSqueak is a USV detection system that relies on deep-learning algorithms. It is capable of human-like USV detection while also allowing for manual corrections in case background noise or faint signals lead to false or missing automatic detections^28^. For the analysis of 40-kHz USVs multidetect function with the default network “Rat Detector YOLO R1.mat.” was used. The detection parameters were set as follows: low-frequency cutoff to 20 kHz, high-frequency cutoff to 120 kHz, and score threshold to 1. Detected calls were manually reviewed in DeepSqueak in a random sample control of four files per time point to ensure reasonable detection quality. Since USV onsets, offsets, and contours in our dataset were reliably assessed, and both the rate of background noise being falsely recognized as calls and the rate of calls not being recognized despite being visible to the experimenter in DeepSqueak, were below 5%, we decided to rely on the automated detection. The following parameters were then extracted and analyzed: number of calls, call length, principal frequency, minimum frequency, maximum frequency, power, sinuosity, and tonality. Call features were averaged across the calls of each animal.

### Elevated plus maze

The elevated plus maze test was performed at P22. The testing apparatus consisted of a plus-shaped maze elevated 82 cm above the ground with four arms (45 cm x 10 cm) connected by a central area (10 cm x 10 cm). Two of the arms at the opposite of each other were surrounded by walls with a height of 38 cm (closed arms), while the others did not have walls (open arms). Lighting conditions were controlled to ensure 210 lx in the central area, 200 lx in the open arms, and 170 lx in the closed arms. Rats were placed in the central part of the arena facing an open arm and allowed to explore the maze freely for 10 min while being automatically tracked by the SYGNIS Tracker software. Connected to a video camera positioned above the maze, this software automatically detects the position and movement of the animals. Then the time spent in open and closed arms, visits in the open arms, and distance traveled in the open arms were then analyzed.

### Open field

The open field paradigm was performed at P26. The animals were placed in alternating corners of a standard open field apparatus (60 cm x 60 cm x 60 cm) facing the walls and allowed to explore the arena freely for 10 min. Light level intensity was set to 250 lx. A video camera was placed above the open field, and SYGNIS Tracker software was used for automated position tracking of the animals. The total distance traveled as well as time spent and distance traveled in the center (15 cm x 15 cm region in the middle of the field) and the time spent in the edges (within 15 cm of the walls) were then analyzed.

### Home-cage monitoring

Between P28 and P33, the animals were monitored for 24 h in a home-cage monitoring system (LABORAS, Metris B.V., Netherlands). Clear Makrolon type III cages (38 cm x 22 cm x 25 cm) were placed on LABORAS carbon fiber plates, which allow measuring the induced forces by different behaviors. The LABORAS software then automatically analyzed the data, transforming specific force and weight distribution patterns into behavioral outcomes^21^. In this study, the time spent with eating, drinking, locomotion, immobility and self-grooming, as well as the number of events of each behavior within 24 h, was analyzed. Animals were housed individually with food and water *ad libitum* and under standard housing conditions in a 12-h light/dark cycle. Before the start of each experiment, cages were calibrated according to the instructions of the manufacturer.

### Gait analysis

At P35, a comprehensive gait analysis was performed using the CatWalk XT system version 10.6 (Noldus, Wageningen, The Netherlands). The experimental procedure was performed as previously described in rats with minor modifications^53^. The walkway width was set to 7.5 cm. Rats were placed in the corridor (130 cm length) and allowed to cross the walkway voluntarily until the completion of three compliant runs. A run was defined as compliant if the rat walked down the walkway without stopping or changing direction. The minimum duration for a run was set to 0.5 s, the maximum to 10 s, and the maximum run variation to 30%. After three compliant runs were acquired, the rat was removed from the walkway and returned to its home cage.

For analysis, the system performed automatic footprint classification, which was manually corrected by an experimenter blinded to genotypes. To generate the different gait parameters, the three runs of each animal were averaged as one trial. Based on previous studies and the interpretability of the data, the following parameters were analyzed: run average speed, run duration, number of steps, number of step patterns, stride length, paw print area, paw print width and length, swing speed, regularity index, support (single, diagonal, lateral, three, and four), width between front and hind paws, cadence, stand time, and swing time^54^. Parameters assessed separately for each of the four paws were averaged across paws for each animal, as paw-specific changes were not expected and to facilitate interpretation of the large dataset.

### Spontaneous alternation T-maze

The unrewarded spontaneous alternation T-maze paradigm was used between P37 and P38 to assess spatial working memory and cognitive flexibility as previously described with minor modifications^30,55^. The T-shaped testing apparatus consisted of three arms (50 cm x 10 cm) attached to a 10 cm x 10 cm central connection area. A distinction is made between a start arm and two goal arms (left and right), which feature guillotine doors at their entrance to allow the experimenter to close the arms when needed. At the start of the test, the rat was placed at the distal end of the start arm facing away from the central area and allowed to freely choose between either the right or the left goal arm. Once the rat had chosen a goal arm, it was confined in this arm for 30 s by closing the guillotine door. A second examiner noted down the chosen arm (R or L) and the decision time, which was defined as the time the animal needed from being placed in the maze to choose an arm by entering it with all four paws and the tail (tail tip criterion). Following this retention interval, the rat was placed at the beginning of the start arm again, facing away from the central area, and the next trial was started. All trials were limited to a maximum duration of 2 min. If an animal failed to complete a trial within this time, it was placed in the last chosen arm for 30 s before the next trial began. These trials were documented by the examiner as “uncompleted trials” and later evaluated in the completion rate parameter. A total of 11 trials were conducted, allowing for a maximum of 10 possible alternations if all trials were completed. The data were analyzed for (1) completion rate (ratio of completed trials to total trials), (2) alternation rate (ratio of successful alternations to possible alternations), and (3) average decision time (sum of all decision times divided by the number of completed trials). Possible alternations were determined based solely on completed trials, meaning that incomplete trials were excluded from this calculation as previously suggested^31^.

### Three-chamber social interaction test

The three-chamber test was performed between P40 and P42. The experimental apparatus consisted of a transparent plexiglass box (90 cm x 60 cm) that was divided into equally sized right, left, and center chambers (30 cm x 60 cm). The compartments were separated by transparent walls with removable doors (12 cm x 10 cm). The right and left chambers each included a cylindrical wire cup (diameter: 11 cm, height: 20 cm). The test consisted of three trials: a habituation, a sociability, and a social novelty trial. In the habituation trial, the wire cups were empty, and the experimental rat was placed in the center chamber, allowing it to freely explore all compartments of the apparatus for 10 min. Then, to test sociability, a completely unfamiliar stimulus rat (Stranger 1) was introduced in one of the wire cups while the other one remained empty. The experimental rat was again allowed to explore the arena freely for 10 min. In the last trial, social novelty was tested by additionally placing a second completely unfamiliar stimulus rat (Stranger 2) in the other wire cup, while Stranger 1 was now already known to the experimental rat. Stimulus rats were sex- and age-matched conspecifics. The positions of Stranger 1 and Stranger 2 were systemically alternated to avoid a side bias. The time spent in proximity to each wire cup (within a concentric area around the wire cup with a diameter of 25 cm) was automatically assessed using a video camera and SYGNIS Tracker software. The sociability index was calculated by dividing the time spent in proximity to Stranger 1 by the time spent in proximity to the empty cup during the second trial. Accordingly, the social novelty index was calculated by dividing the time spent in proximity to Stranger 2 by the time spent in proximity to Stranger 1 during the third trial.

### Direct social interaction test

The direct social interaction paradigm was performed between P44 and P46. As described in previous studies, subject animals were single housed overnight in standard Makrolon type III rat cages without enrichment to increase social motivation^19,56^. On the following day, testing was conducted in two phases. The first 5 min served as a habituation phase, in which the experimental rat was placed alone in an open field arena (60 cm x 60 cm x 60 cm). Then immediately afterward, the second phase was initiated by placing a completely unfamiliar, sex- and age-matched stimulus rat next to the experimental rat to allow free social interaction and exploration of the environment. The behavior was captured for 10 min using a video camera placed above the open field. Subject rats and stimulus rats were marked on their backs with two different colors (green and red, respectively) to differentiate them.

Social and non-social behaviors of the experimental rat were then manually scored by an observer blinded to genotypes using the open-source event-logging software BORIS^57^. Scoring of social behaviors included anogenital sniffing, (non-anogenital) body sniffing, crawling over/under the stimulus rat, play behavior (including neck biting, pinning, and wrestling), following behavior, and nose-to-nose contact. As non-social behaviors, rearing and self-grooming were scored. Social behaviors were only scored if initiated and conducted by the experimental rat (e.g., it was only counted as “following behavior” if the experimental rat was following the stimulus rat, not vice versa). The duration of each behavior during the 10 min of social interaction was extracted from BORIS and analyzed. Additionally, the total duration of social behaviors and non-social behaviors was calculated.

### Socially induced ultrasonic vocalizations

USVs during the direct social interaction paradigm were measured using an UltraSoundGate condenser microphone (CM16/CMPAc) that was placed approximately 60 cm above the open field arena. The subsequent experimental setup and recording parameters are in accordance with the methodology used in this study for USV recordings at pup age.

Recordings were analyzed using DeepSqueak (Version 3.1.0) in MATLAB version R2024a. 50-kHz USVs were automatically detected using the multidetect function, with the default network “Rat Detector YOLO R1.mat.”. The detection parameters were set as follows: low-frequency cutoff to 35 kHz, high-frequency cutoff to 120 kHz, and score threshold to 1. However, going through the scored files, we noticed inconsistencies in the automated scoring quality that are most likely attributable to varying positions of the rats throughout the arena and the fact that, in contrast to the isolation-induced USVs, recordings did not take place in a sound-attenuated chamber with a closed top, and consequently background noises were more prevalent. All files were therefore manually corrected in DeepSqueak by an experimenter blinded to genotypes. This included accepting correctly detected USVs, rejecting false positives, adding false negatives, and resizing detection boxes if needed.

The following parameters were then extracted and analyzed: number of calls, call length, principal frequency, minimum frequency, maximum frequency, sinuosity, and tonality. Call features were averaged for the individual calls of each animal.

As described in previous studies, 22-kHz calls were observed only occasionally during direct social interaction^56,58^, likely due to the absence of an aversive stimulus. Thus, a detailed analysis of this call category was not conducted.

### Statistical analysis and plots

Statistical analyses were performed using GraphPad Prism 10 (GraphPad Software, La Jolla, CA, USA). This study was designed and analyzed as an exploratory investigation to broadly characterize behavioral phenotypes in *Magel2*^*Pmut*^ rats. Outliers were excluded based on the ROUT method (Q = 1%), applied within each genotype and sex subgroup prior to any further analysis. Normality of residuals was not formally tested using hypothesis-based methods such as the Shapiro-Wilk test, which can have limited power in small samples and may flag trivial deviations in larger ones^59,60^. Instead, Q–Q plots were visually inspected and discussed with a statistician. As no strong deviations from normality were observed, parametric methods were deemed appropriate. A two-way ANOVA with genotype and sex as factors, including their interaction, was performed for each behavioral outcome. *Post hoc* unpaired, two-sided t-tests with Bonferroni correction were conducted only when a significant genotype-by-sex interaction was present to assess genotype effects within each sex, as recommended in the literature^61^. In these cases, the results were visualized for each sex separately. Otherwise, main effects of genotype from the ANOVA were interpreted and visualized as follows: both sexes were displayed together in a single boxplot per genotype group, with data points distinguished by color shading (males in darker shades, females in lighter shades). This facilitates visual distinction between sexes, consistent with approaches used in previous studies^19,62^. Body weight development was presented as a time-dependent curve. Since displaying individual data points in this context would have reduced the clarity of the figure, sexes were instead shown separately in Supplementary Fig. 1. P values of *P* < 0.05 were considered to be statistically significant. A detailed presentation of all performed statistics, including outlier analysis and exact P values, can be found in the supplementary material (Supplementary Tables S1-23). Box-and-whisker plots were generated using standard conventions, with the box representing the interquartile range, the central line indicating the median, and whiskers extending to the minimum and maximum values of the data.

## Supplementary Information

Below is the link to the electronic supplementary material.

Supplementary Material 1

## Data Availability

The DNA sequences generated and analyzed during the current study using Sanger sequencing are available in the DNA Data Bank of Japan (DDBJ) repository [Accession numbers: LC884486, LC884487, LC884488, and LC884489]. The rodent behavioral testing datasets generated and analyzed during the current study are available from the corresponding author on reasonable request.

## References

[1] Schubert, T. & Schaaf, C. P. MAGEL2 (patho-)physiology and Schaaf–Yang syndrome. *Dev. Med. Child. Neurol.* **67**, 35–48 (2025). 38950199 10.1111/dmcn.16018 PMC11625468

[2] Tacer, K. F. & Potts, P. R. Cellular and disease functions of the Prader–Willi syndrome gene MAGEL2. *Biochem. J.* **474**, 2177–2190 (2017). 28626083 10.1042/BCJ20160616 PMC5594744

[3] Gregory, L. C. et al. Mutations in MAGEL2 and L1CAM are associated with congenital hypopituitarism and arthrogryposis. *J. Clin. Endocrinol. Metabolism*. **104**, 5737–5750 (2019). 10.1210/jc.2019-00631 PMC6916815 31504653

[4] Lee, S. Expression and imprinting of MAGEL2 suggest a role in Prader-willi syndrome and the homologous murine imprinting phenotype. *Hum. Mol. Genet.* **9**, 1813–1819 (2000). 10915770 10.1093/hmg/9.12.1813

[5] Goel, M. et al. Integrative functions of the hypothalamus: linking cognition, emotion and physiology for well-being and adaptability. *Annals Neurosciences* **32**(2), (2024). 10.1177/09727531241255492 PMC11559822 39544638

[6] Schaaf, C. P. et al. Truncating mutations of MAGEL2 cause Prader-Willi phenotypes and autism. *Nat. Genet.* **45**, 1405–1408 (2013). 24076603 10.1038/ng.2776 PMC3819162

[7] Patak, J. et al. MAGEL2-related disorders: A study and case series. *Clin. Genet.* **96**, 493–505 (2019). 31397880 10.1111/cge.13620 PMC6864226

[8] Boccaccio, I. et al. The human Magel2 gene and its mouse homologue are paternally expressed and mapped to the prader-willi region. *Hum. Mol. Genet.* **8**, 2497–2505 (1999). 10556298 10.1093/hmg/8.13.2497

[9] Angulo, M. A., Butler, M. G. & Cataletto, M. E. Prader-Willi syndrome: a review of clinical, genetic, and endocrine findings. *J. Endocrinol. Investig.* **38**, 1249–1263 (2015). 26062517 10.1007/s40618-015-0312-9 PMC4630255

[10] Marbach, F. et al. The adult phenotype of Schaaf-Yang syndrome. *Orphanet J. Rare Diseases* **15** (2020). 10.1186/s13023-020-01557-8 PMC7574436 33076953

[11] Schaaf, C. P. et al. in GeneReviews. (eds. M.P. Adam (Seattle (WA); (2021).

[12] Mccarthy, J. et al. Schaaf-Yang syndrome overview: report of 78 individuals. *Am. J. Med. Genet. Part. A*. **176**, 2564–2574 (2018). 30302899 10.1002/ajmg.a.40650 PMC6585857

[13] Kanber, D. et al. A paternal deletion of MKRN3, MAGEL2 and NDN does not result in Prader–Willi syndrome. *Eur. J. Hum. Genet.* **17**, 582–590 (2009). 19066619 10.1038/ejhg.2008.232 PMC2986273

[14] Buiting, K. et al. Clinical phenotypes of MAGEL2 mutations and deletions. *Orphanet J. Rare Dis.* **9**, 40 (2014). 24661356 10.1186/1750-1172-9-40 PMC3987887

[15] Centeno-Pla, M. et al. Subcellular localisation of truncated MAGEL2 proteins: insight into the molecular pathology of Schaaf-Yang syndrome. *J. Med. Genet.* **61**, 780–782 (2024). 38548315 10.1136/jmg-2024-109898

[16] Heimdörfer, D. et al. Truncated variants of MAGEL2 are involved in the etiologies of the Schaaf-Yang and Prader-Willi syndromes. *Am. J. Hum. Genet.* **111**, 1383–1404 (2024). 38908375 10.1016/j.ajhg.2024.05.023 PMC11267527

[17] Dötsch, L., Matesevac, L., Strong, T. V. & Schaaf, C. P. Caregiver-based perception of disease burden in Schaaf‐Yang syndrome. *Molecular Genet. & Genomic Medicine* **11** (2023). 10.1002/mgg3.2262 PMC10724517 37533374

[18] Bervini, S. & Herzog, H. Mouse models of Prader-willi syndrome: a systematic review. *Front. Neuroendocrinol.* **34**, 107–119 (2013). 23391702 10.1016/j.yfrne.2013.01.002

[19] Reznik, D. L. et al. Magel2 Truncation alters select behavioral and physiological outcomes in a rat model of Schaaf-Yang syndrome. *Disease Models & Mechanisms* **16**(2) (2023). 10.1242/dmm.049829 PMC9922728 36637363

[20] Ellenbroek, B. & Youn, J. Rodent models in neuroscience research: is it a rat race? *Dis. Model. Mech.* **9**, 1079–1087 (2016). 27736744 10.1242/dmm.026120 PMC5087838

[21] Quinn, L. P. et al. Initial Pharmacological validation of a system allowing continuous monitoring of laboratory rodent behaviour. *J. Neurosci. Methods*. **130**, 83–92 (2003). 14583407 10.1016/s0165-0270(03)00227-9

[22] Garrick, J. M., Costa, L. G., Cole, T. B. & Marsillach, J. Evaluating gait and locomotion in rodents with the catwalk. *Current Protocols* **1** (2021). 10.1002/cpz1.220 PMC8363132 34370398

[23] Premoli, M., Pietropaolo, S., Wöhr, M., Simola, N. & Bonini, S. A. Mouse and rat ultrasonic vocalizations in neuroscience and neuropharmacology: state of the Art and future applications. *Eur. J. Neurosci.* **57**, 2062–2096 (2023). 36889803 10.1111/ejn.15957

[24] Möhrle, D. et al. Characterizing maternal isolation-induced ultrasonic vocalizations in a gene–environment interaction rat model for autism. *Genes Brain Behavior* **22**(3) (2023). 10.1111/gbb.12841 PMC10242206 36751016

[25] Berg, E. L. et al. Translational outcomes relevant to neurodevelopmental disorders following early life exposure of rats to Chlorpyrifos. *Journal Neurodevelopmental Disorders* **12**, 40 (2020). 10.1186/s11689-020-09342-1 PMC7745485 33327943

[26] Hoffmeister, J. D., Kelm-Nelson, C. A. & Ciucci, M. R. Manipulation of vocal communication and anxiety through Pharmacologic modulation of norepinephrine in the Pink1-/- rat model of Parkinson disease. *Behav. Brain Res.* **418**, 113642 (2022). 34755639 10.1016/j.bbr.2021.113642 PMC8671235

[27] Lenell, C., Broadfoot, C. K., Schaen-Heacock, N. E. & Ciucci, M. R. Biological and acoustic sex differences in rat ultrasonic vocalization. *Brain Sci.* **11**, 459 (2021). 33916537 10.3390/brainsci11040459 PMC8067311

[28] Coffey, K. R., Marx, R. E. & Neumaier, J. F. DeepSqueak: a deep learning-based system for detection and analysis of ultrasonic vocalizations. *Neuropsychopharmacology* **44**, 859–868 (2019). 30610191 10.1038/s41386-018-0303-6 PMC6461910

[29] Kappos, E. A. et al. Validity and reliability of the catwalk system as a static and dynamic gait analysis tool for the assessment of functional nerve recovery in small animal models. *Brain Behav.* **7**, e00723 (2017). 28729931 10.1002/brb3.723 PMC5516599

[30] Deacon, R. M. J. & Rawlins J.N.P. T-maze alternation in the rodent. *Nat. Protoc.* **1**, 7–12 (2006). 17406205 10.1038/nprot.2006.2

[31] Hetzer, S. M. et al. Early measures of TBI severity poorly predict later individual impairment in a rat fluid percussion model. *Brain Sci.* **13**, 1230 (2023). 37759831 10.3390/brainsci13091230 PMC10526292

[32] Thomason, M. M. et al. Neurocognitive and neurobehavioral phenotype of youth with Schaaf-Yang syndrome. *J. Autism Dev. Disord.* **50**, 2491–2500 (2020). 30343463 10.1007/s10803-018-3775-7

[33] Zeskind, P. S. et al. Development of translational methods in spectral analysis of human infant crying and rat pup ultrasonic vocalizations for early neurobehavioral assessment. *Front. Psychiatry*. **2**, 56 (2011). 22028695 10.3389/fpsyt.2011.00056 PMC3199610

[34] Zeskind, P. S. et al. Translational analysis of effects of prenatal cocaine exposure on human infant cries and rat pup ultrasonic vocalizations. *PLoS ONE*. **9**, e110349 (2014). 25338015 10.1371/journal.pone.0110349 PMC4206414

[35] Kim, K. C. et al. Male-specific alteration in excitatory post‐synaptic development and social interaction in pre‐natal valproic acid exposure model of autism spectrum disorder. *J. Neurochem.* **124**, 832–843 (2013). 23311691 10.1111/jnc.12147

[36] Dawson, M. S. et al. Sexual dimorphism in the social behaviour of Cntnap2-null mice correlates with disrupted synaptic connectivity and increased microglial activity in the anterior cingulate cortex. *Communications Biology* **6**, 846 (2023). 10.1038/s42003-023-05215-0 PMC10427688 37582968

[37] Yin, J. & Schaaf, C. P. Autism genetics – an overview. *Prenat. Diagn.* **37**, 14–30 (2017). 27743394 10.1002/pd.4942

[38] Spear, L. P., Brake, S. C. & Periadolescence Age-dependent behavior and psychopharmacological responsivity in rats. *Dev. Psychobiol.* **16**, 83–109 (1983). 6339302 10.1002/dev.420160203

[39] John, S. R. et al. Simultaneous recording of ultrasonic vocalizations and sniffing from socially interacting individual rats using a miniature microphone. *Cell. Rep. Methods*. **3**, 100638 (2023). 37939710 10.1016/j.crmeth.2023.100638 PMC10694494

[40] Braun, A. A., Skelton, M. R., Vorhees, C. V. & Williams, M. T. Comparison of the elevated plus and elevated zero mazes in treated and untreated male Sprague–Dawley rats: effects of anxiolytic and anxiogenic agents. *Pharmacol. Biochem. Behav.* **97**, 406–415 (2011). 20869983 10.1016/j.pbb.2010.09.013 PMC3006066

[41] Ramos, A. Animal models of anxiety: do I need multiple tests? *Trends Pharmacol. Sci.* **29**, 493–498 (2008). 18755516 10.1016/j.tips.2008.07.005

[42] Fountain, M. D. et al. The phenotypic spectrum of Schaaf-Yang syndrome: 18 new affected individuals from 14 families. *Genet. Sci.* **19**, 45–52 (2017). 10.1038/gim.2016.53 PMC5116288 27195816

[43] Chen, Y. J. et al. Detection of subtle neurological alterations by the catwalk XT gait analysis system. *J. Neuroeng. Rehabil.* **11**, 62 (2014). 24739213 10.1186/1743-0003-11-62 PMC3997750

[44] Hebach, N. R. et al. A retrospective analysis of growth hormone therapy in children with Schaaf–Yang syndrome. *Clin. Genet.* **100**, 298–307 (2021). 34013972 10.1111/cge.14000

[45] Fountain, M. D., Tao, H., Chen, C. A., Yin, J. & Schaaf, C. P. Magel2 knockout mice manifest altered social phenotypes and a deficit in preference for social novelty. *Genes Brain Behav.* **16**, 592–600 (2017). 28296079 10.1111/gbb.12378 PMC5495607

[46] Wieting, J., Jahn, K., Bleich, S., Deest, M. & Frieling, H. Sex differences in MAGEL2 gene promoter methylation in high functioning autism - trends from a pilot study using nanopore Cas9 targeted long read sequencing. *BMC Med. Genomics* **17**, 279 (2024). 10.1186/s12920-024-02053-9 PMC11606058 39609859

[47] Mcgraw, C. M., Ward, C. S. & Samaco, R. C. Genetic rodent models of brain disorders: perspectives on experimental approaches and therapeutic strategies. *Am. J. Med. Genet. Part. C: Seminars Med. Genet.* **175**, 368–379 (2017). 10.1002/ajmg.c.31570 PMC5659732 28910526

[48] Hãnell, A. & Marklund, N. Structured evaluation of rodent behavioral tests used in drug discovery research. *Frontiers Behav. Neuroscience* **8** (2014). 10.3389/fnbeh.2014.00252 PMC4106406 25100962

[49] Bosque Ortiz, G. M., Santana, G. M. & Dietrich, M. O. Deficiency of the paternally inherited gene Magel2 alters the development of separation-induced vocalization and maternal behavior in mice. *Genes Brain Behavior* **21**(1) (2022). 10.1111/gbb.12776 PMC9744533 34812568

[50] Bischof, J. M., Stewart, C. L. & Wevrick, R. Inactivation of the mouse Magel2 gene results in growth abnormalities similar to Prader-Willi syndrome. *Hum. Mol. Genet.* **16**, 2713–2719 (2007). 17728320 10.1093/hmg/ddm225

[51] Althammer, F. et al. Analysis of the hypothalamic Oxytocin system and Oxytocin receptor-expressing astrocytes in a mouse model of Prader–Willi syndrome. *Journal Neuroendocrinology* **34**(12) (2022). 10.1111/jne.13217 36458331

[52] Herdt, R. et al. Enhancing the analysis of murine neonatal ultrasonic vocalizations: Development, evaluation, and application of different mathematical models. *J. Acoust. Soc. Am.* **156**, 2448–2466 (2024). 39400270 10.1121/10.0030473

[53] Ye, H. et al. Curcumin regulates autophagy through SIRT3-SOD2-ROS signaling pathway to improve quadriceps femoris muscle atrophy in KOA rat model. *Scientific Reports* **14**, 8176 (2024). 10.1038/s41598-024-58375-2 PMC11001965 38589505

[54] Timotius, I. K. et al. CatWalk XT gait parameters: a review of reported parameters in pre-clinical studies of multiple central nervous system and peripheral nervous system disease models. *Frontiers Behav. Neuroscience* **17** (2023). 10.3389/fnbeh.2023.1147784 PMC10284348 37351154

[55] Friedman, L. K., Peng, H. & Zeman, R. J. Cannabidiol reduces lesion volume and restores vestibulomotor and cognitive function following moderately severe traumatic brain injury. *Exp. Neurol.* **346**, 113844 (2021). 34428457 10.1016/j.expneurol.2021.113844

[56] Redecker, T. M., Kisko, T. M., Schwarting, R. K. W. & Wohr, M. Effects of Cacna1c haploinsufficiency on social interaction behavior and 50-kHz ultrasonic vocalizations in adult female rats. *Behav. Brain Res.* **367**, 35–52 (2019). 30902660 10.1016/j.bbr.2019.03.032

[57] Friard, O. & Gamba, M. BORIS: a free, versatile open-source event‐logging software for video/audio coding and live observations. *Methods Ecol. Evol.* **7**, 1325–1330 (2016).

[58] Gzielo, K. et al. Valproic acid exposure impairs ultrasonic communication in infant, adolescent and adult rats. *Eur. Neuropsychopharmacol.* **41**, 52–62 (2020). 32978035 10.1016/j.euroneuro.2020.09.006

[59] Ruxton, G. D., Wilkinson, D. M. & Neuhäuser, M. Advice on testing the null hypothesis that a sample is drawn from a normal distribution. *Anim. Behav.* **107**, 249–252 (2015).

[60] Gosselin, R. D. Guidelines on statistics for researchers using laboratory animals: the essentials. *Lab. Anim.* **53**, 28–42 (2019). 29954248 10.1177/0023677218783223

[61] Phillips, B., Haschler, T. N. & Karp, N. A. Statistical simulations show that scientists need not increase overall sample size by default when including both sexes in in vivo studies. *PLoS Biol.* **21**, e3002129 (2023). 37289836 10.1371/journal.pbio.3002129 PMC10284409

[62] Eltokhi, A., Kurpiers, B. & Pitzer, C. Behavioral tests assessing neuropsychiatric phenotypes in adolescent mice reveal strain- and sex-specific effects. *Scientific Reports* **10**, 11263 (2020). 10.1038/s41598-020-67758-0 PMC7347854 32647155

