# Supplementary material for: In-depth behavioral characterization of a rat model of Schaaf-Yang syndrome
Source: Sci Rep. 2025 Oct 30;15:37929. doi: 10.1038/s41598-025-20958-y (PMC12575713; doi:10.1038/s41598-025-20958-y)
Supplement: Supplementary file 1 — Supplementary Material 1 [file 41598_2025_20958_MOESM1_ESM.pdf]

In-depth behavioral characterization of a rat model of Schaaf-Yang syndrome: Supplementary Figures

Felix Franke[1], Semih Ertürk[1], Johann G. Maass[1,2], Dominik Kamionek[1], Tim Schubert[1,3], Claudia Pitzer[4], Susanne Theiß[1], Christine Fischer[1], Rachel B. Gilmore[1], Eva Dwornicki[1], Colleen R. Bocke[5], Gina L. C. Yosten[5], Christian P. Schaaf[1], Ferdinand Althammer[1*]

[1] *Institute of Human Genetics, Heidelberg University, Heidelberg, Germany*
[2] *Division of Genetics and Genomics, Boston Children's Hospital, Boston, Massachusetts, United States*
[3] *Department of* Applied Mathematics and Theoretical Physics, Cambridge University, Cambridge, United Kingdom
[4] *Interdisciplinary Neurobehavioral Core, Heidelberg University, Heidelberg, Germany*
[5] *Department of Pharmacology and Physiology, Saint Louis University School of Medicine, Saint Louis, Missouri, United States*
[*] *Corresponding author: Ferdinand Althammer (ferdinand.althammer@med.uni-heidelberg.de)*

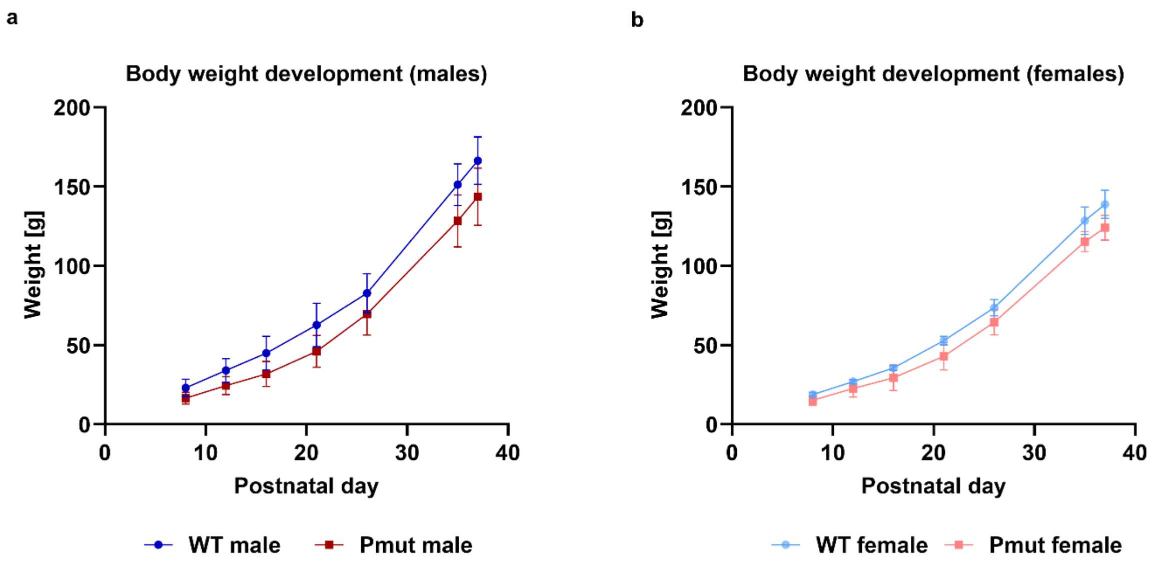

Supplementary Figure S1. Sex-specific visualization of body weight development in *Magel2*^{Pmut} rats and wild-type littermates. (**a**) Body weight development in male wild-type and *Magel2*^{Pmut} rats. (**b**) Body weight development in female wild-type and *Magel2*^{Pmut} rats. No statistical comparisons were performed within sex groups, as two-way ANOVA revealed no significant genotype-by-sex interaction at any time point; therefore, genotype effects were interpreted across sexes. Statistical details are provided in Supplementary Tables S1, S2.

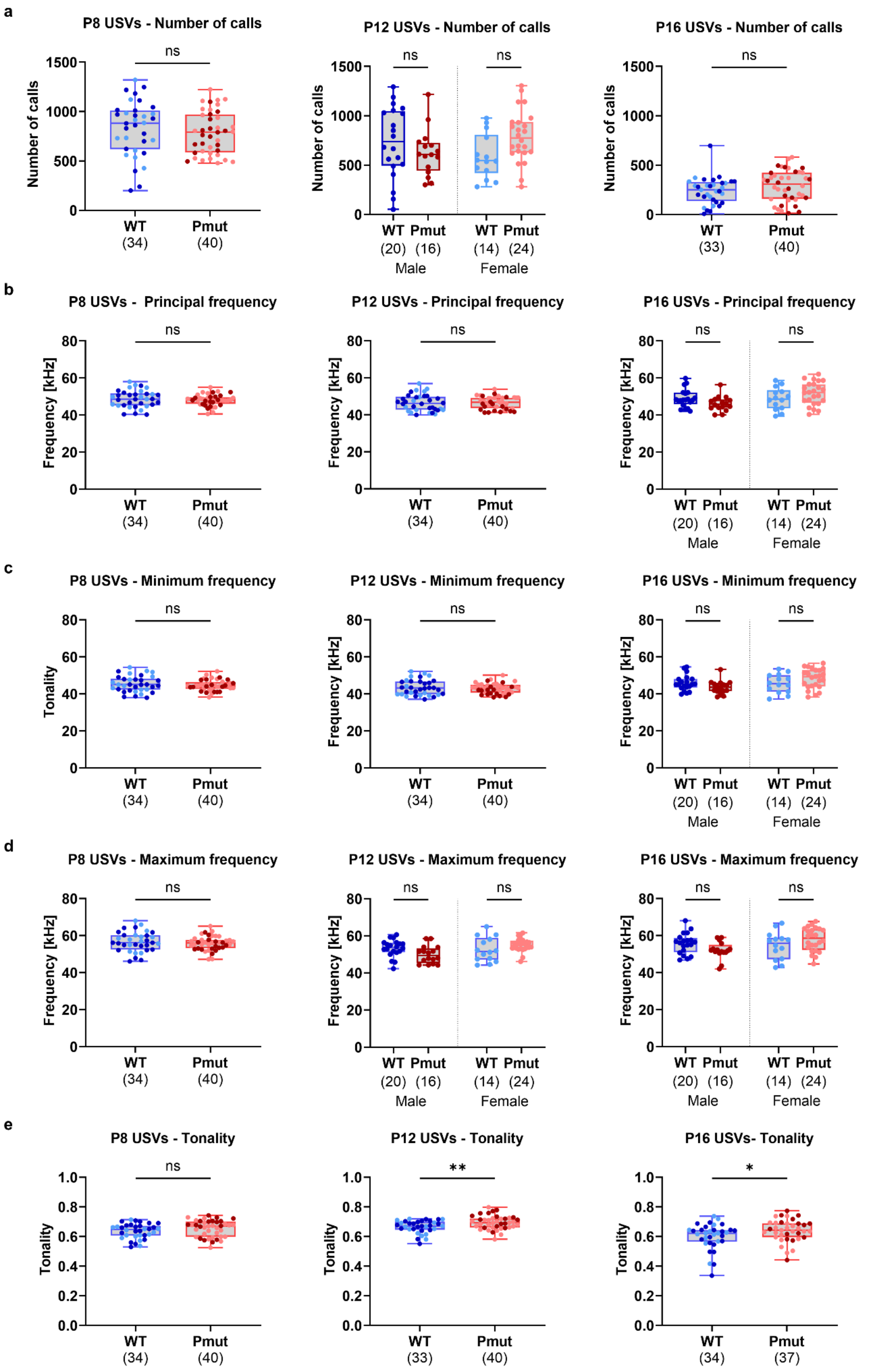
a
P8 USVs - Number of calls
ns
Number of calls
WT (34)
Pmut (40)
P12 USVs - Number of calls
ns
ns
WT Pmut (20) (16) Male
WT Pmut (14) (24) Female
P16 USVs - Number of calls
WT (33)
Pmut (40)
b
P8 USVs - Principal frequency
Frequency [kHz]
P12 USVs - Principal frequency
P16 USVs - Principal frequency
c
P8 USVs - Minimum frequency
Tonality
P12 USVs - Minimum frequency
P16 USVs - Minimum frequency
d
P8 USVs - Maximum frequency
P12 USVs - Maximum frequency
P16 USVs - Maximum frequency
e
P8 USVs - Tonality
P12 USVs - Tonality
**
WT (33)
Pmut (40)
P16 USVs- Tonality
*
WT (34)
Pmut (37)

Supplementary Figure S2. Additional parameters of isolation-induced ultrasonic vocalizations (USVs) in *Magel2*^{Pmut} rats compared to wild-type littermates. (**a**) No significant genotype effects were observed regarding the number of calls at P8 and P16. At P12, a significant genotype-by-sex interaction was detected, but *post hoc* analyses within each sex did not reveal significant genotype differences. (**b-d**) No significant genotype effects were observed for the mean principal, minimum, or maximum frequency of USVs at any time points. While in some cases significant genotype-by-sex interactions were detected, *post hoc* analyses within each sex also did not reveal significant genotype differences. (**e**) USVs of *Magel2*^{Pmut} rats showed significantly increased tonality at P12 and P16. Call features represent averages across all calls of each animal. Sample sizes after exclusion of outliers based on the ROUT method (Q = 1%) are indicated below the graphs. Data points for wild-type animals are shown in blue, and for *Magel2*^{Pmut} animals in red. Within each genotype, darker shades represent males, and lighter shades represent females. ns, not significant; two-way ANOVA with genotype and sex as factors, followed by *post hoc* analyses within each sex when a significant genotype-by-sex interaction was detected. Statistical details are provided in Supplementary Tables S3–S5.

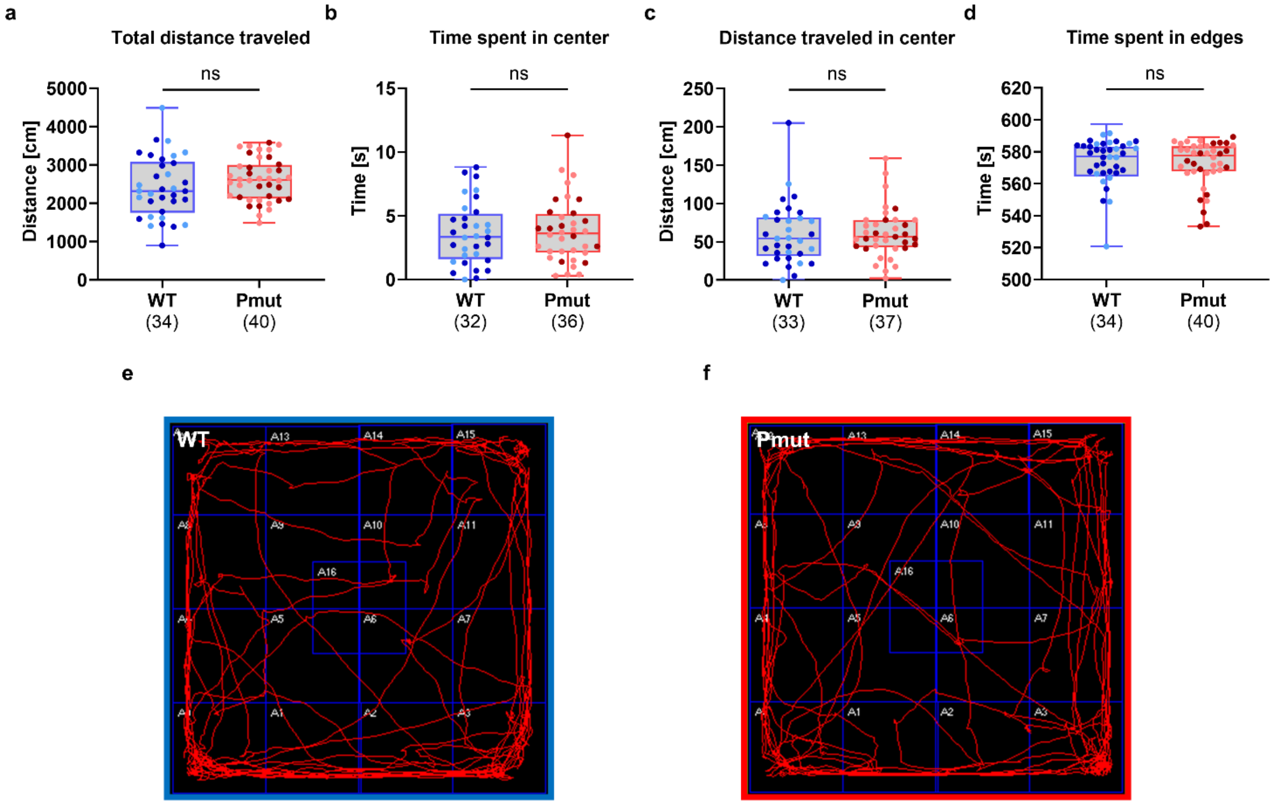

Supplementary Figure S3. Spontaneous locomotion and anxiety-like behavior measured by the open field test in *Magel2*^{Pmut} rats compared to wild-type littermates. (**a–d**) No significant genotype effects were observed for any of the open field parameters analyzed, including total distance traveled, time spent in center, distance traveled in center, and time spent in edges. Sample sizes after exclusion of outliers based on the ROUT method (Q = 1%) are indicated below the graphs. Data points for wild-type animals are shown in blue, and for *Magel2*^{Pmut} animals in red. Within each genotype, darker shades represent males, and lighter shades represent females. ns, not significant; two-way ANOVA with genotype and sex as factors. Statistical details are provided in Supplementary Tables S8, S9. (**e–f**)

Representative examples of track maps in the open field for wild-type and *Magel2*^{Pmut} animals. No apparent differences are observed.

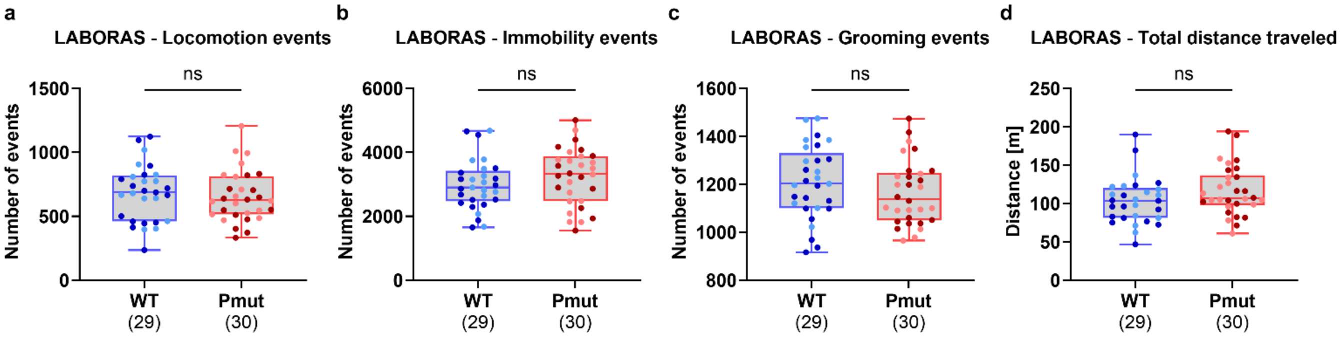

Supplementary Figure S4. Additional parameters of home-cage monitoring using the LABORAS system in *Magel2*^{Pmut} rats compared to wild-type littermates. (**a–c**) No significant genotype effects were observed for the number of locomotion events, immobility events, or grooming events. Sample sizes after exclusion of outliers based on the ROUT method (Q = 1%) are indicated below the graphs. Data points for wild-type animals are shown in blue, and for *Magel2*^{Pmut} animals in red. Within each genotype, darker shades represent males, and lighter shades represent females. ns, not significant; two-way ANOVA with genotype and sex as factors. Statistical details are provided in Supplementary Tables S10, S11.

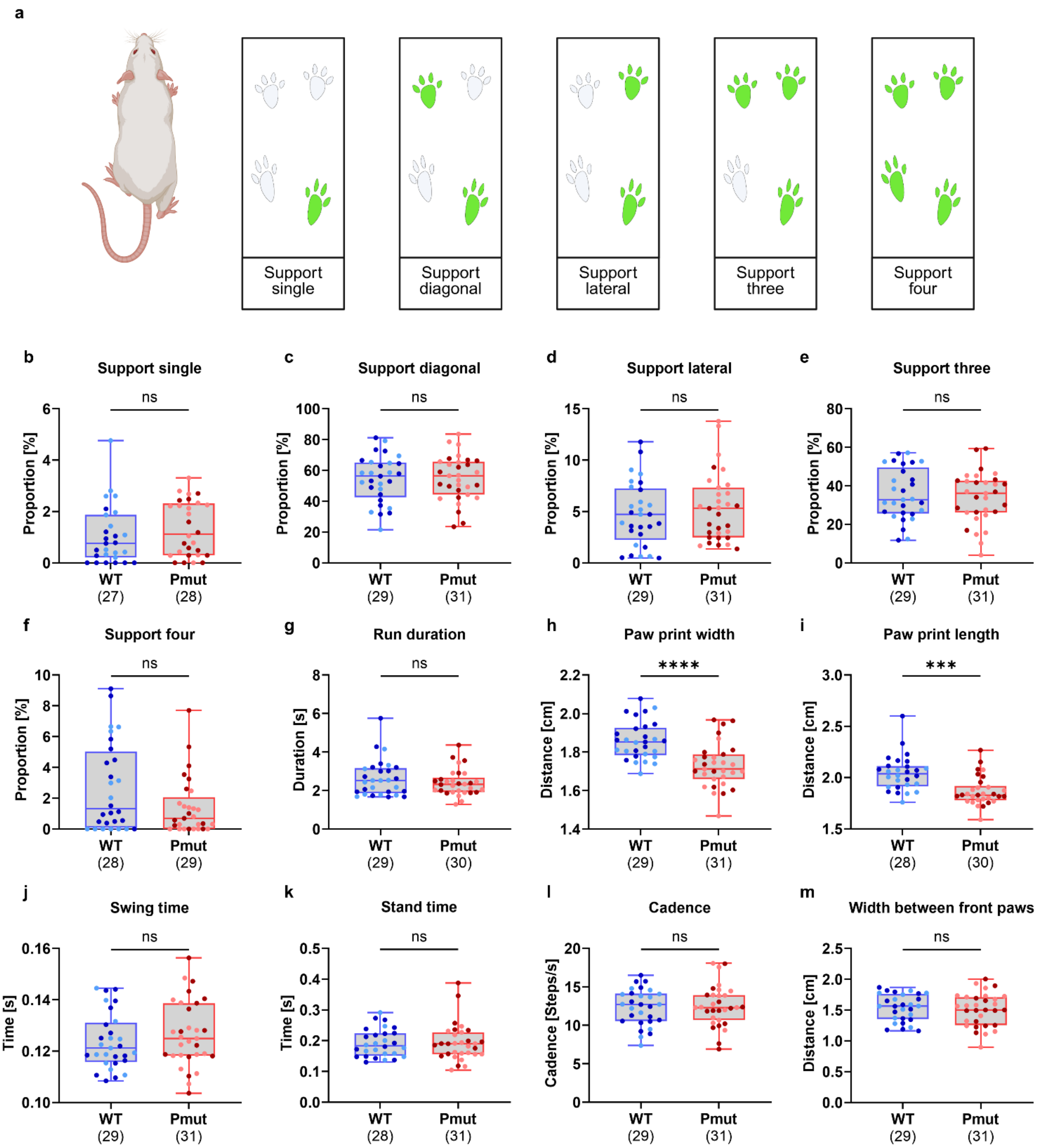

Supplementary Figure S5. Additional parameters of CatWalk XT gait analysis in *Magel2*^{Pmut} rats compared to wild-type littermates. (**a**) Illustration of analyzed support parameters. (**b–f**) No significant genotype effects were observed for any of the support parameters analyzed. (**g**) Run duration was comparable between genotypes. (**h, i**) Paw print width and length were significantly reduced in *Magel2*^{Pmut} rats compared to wild-type littermates. (**j–m**) No significant genotype effects were observed for swing time, stand time, cadence, and width between front paws. Sample sizes after exclusion of outliers based on the ROUT method (Q = 1%) are indicated below the graphs. Data points for wild-type animals are shown in blue, and for *Magel2*^{Pmut} animals in red. Within each genotype, darker shades represent males, and lighter shades represent females. ***P<0.001, ****P<0.0001; ns: not

significant; two-way ANOVA with genotype and sex as factors. Statistical details are provided in Supplementary Tables S12–S14.

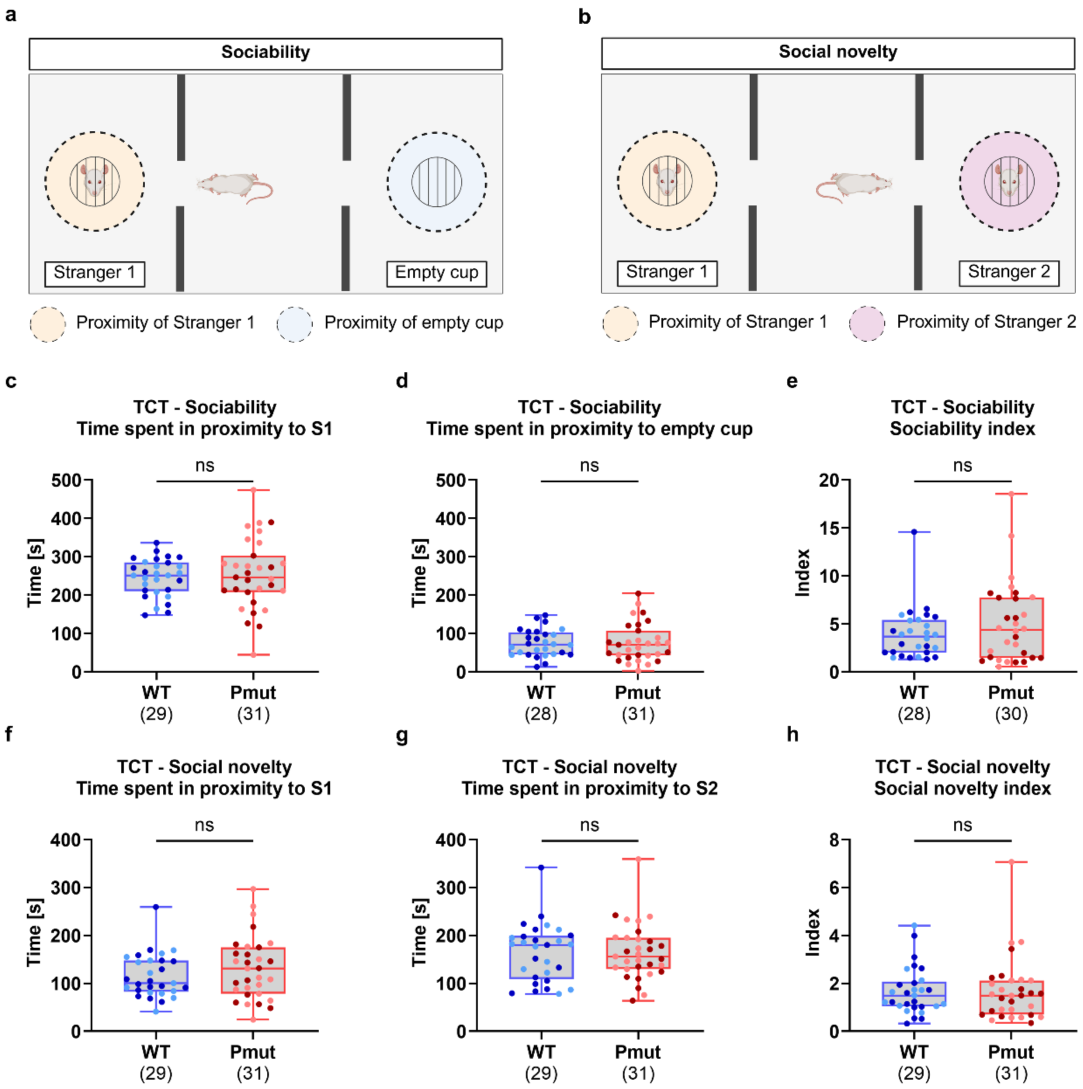

Supplementary Figure S6. Sociability and social novelty as measured by the three-chamber social interaction test in *Magel2*^{Pmut} rats compared to wild-type littermates. (**a–b**) Schematic Illustrations of the two analyzed trials. In the sociability trial, rats typically prefer to spend time in proximity to the unfamiliar Stranger 1 (S1) instead of the empty cup. In the social novelty trial, rats typically prefer to spend time in proximity to the unfamiliar Stranger 2 (S2) instead of the familiar Stranger 1 (S1). (**c–h**) No significant genotype effects were observed for any of the analyzed parameters, including the respective time spent in proximity to each pencil cup and the sociability and social novelty indices. Sample sizes after exclusion of outliers based on the ROUT method (Q = 1%) are indicated below the graphs. Data points for wild-type animals are shown in blue, and for *Magel2*^{Pmut} animals in red. Within each genotype, darker shades represent males, and lighter shades represent females. ns, not significant; two-way ANOVA with genotype and sex as factors. Statistical details are provided in Supplementary Tables S17, S18.

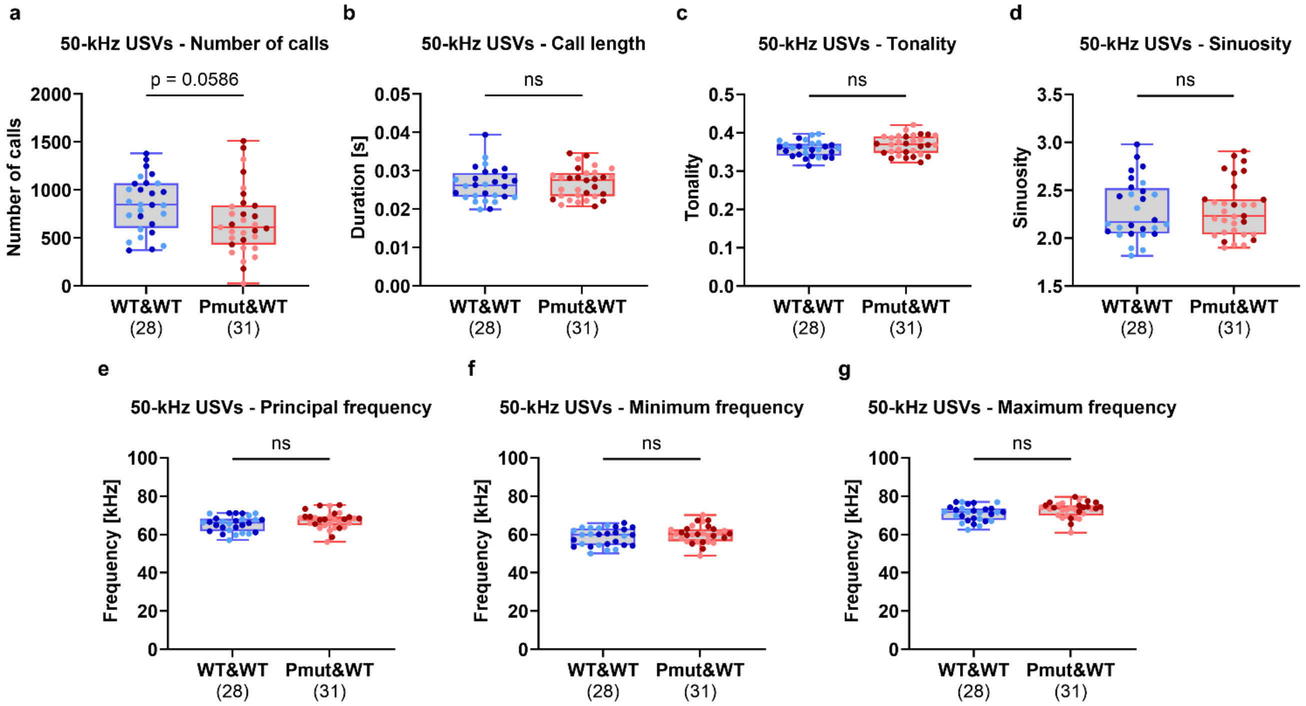

Supplementary Figure S7. 50-kHz ultrasonic vocalizations (USVs) emitted during social interaction in pairings of *Magel2*^{Pmut} rats and wild-type stimulus rats compared to pairings of wild-type littermates and wild-type stimulus rats. (**a–g**) No significant genotype effects were observed for any of the analyzed parameters, including the number of calls, call length, tonality, sinuosity, principal frequency, minimum frequency, and maximum frequency. A trend toward a reduction in the number of calls in pairings including *Magel2*^{Pmut} rats was observed. Call features represent averages across all calls of each animal. Sample sizes after exclusion of outliers based on the ROUT method (Q = 1%) are indicated below the graphs. Data points for pairings of wild-type littermates and wild-type stimulus rats are shown in blue, and for pairings of *Magel2*^{Pmut} rats and wild-type stimulus rats in red. Within each pairing, darker shades represent males, and lighter shades represent females. ns, not significant; two-way ANOVA with genotype and sex as factors. Statistical details are provided in Supplementary Tables S22, S23.

In-depth behavioral characterization of a rat model of Schaaf-Yang syndrome: Supplementary Tables

Felix Franke[1], Semih Ertürk[1], Johann G. Maass[1,2], Dominik Kamionek[1], Tim Schubert[1,3], Claudia Pitzer[4], Susanne Theiß[1], Christine Fischer[1], Rachel B. Gilmore[1], Eva Dwornicki[1], Colleen R. Bocke[5], Gina L. C. Yosten[5], Christian P. Schaaf[1], Ferdinand Althammer[1*]

[1] *Institute of Human Genetics, Heidelberg University, Heidelberg, Germany*
[2] *Division of Genetics and Genomics, Boston Children's Hospital, Boston, Massachusetts, United States*
[3] *Department of* Applied Mathematics and Theoretical Physics, Cambridge University, Cambridge, United Kingdom
[4] *Interdisciplinary Neurobehavioral Core, Heidelberg University, Heidelberg, Germany*
[5] *Department of Pharmacology and Physiology, Saint Louis University School of Medicine, Saint Louis, Missouri, United States*
[*] *Corresponding author: Ferdinand Althammer (ferdinand.althammer@med.uni-heidelberg.de)*

Figure	Parameter	Sample size before outlier analysis				Outliers removed (ROUT, Q = 1%)	Sample size after outlier analysis			
		WT male	WT female	Pmut male	Pmut female		WT male	WT female	Pmut male	Pmut female
Fig. 1c	P8 Weight	14	10	10	14	1	14	9	10	14
Fig. 1c	P12 Weight	14	10	10	14	1	14	9	10	14
Fig. 1c	P16 Weight	14	10	10	14	1	14	9	10	14
Fig. 1c	P21 Weight	20	14	16	24	1	20	13	16	24
Fig. 1c	P26 Weight	20	14	16	24	4	18	13	16	23
Fig. 1c	P35 Weight	16	13	14	17	1	16	13	14	16
Fig. 1c	P37 Weight	16	9	14	15	0	16	9	14	15

Supplementary Table S1. Sample size before and after outlier analysis (ROUT, Q = 1%) for body weight assessment in *Magel2*^{Pmut} rats and wild-type controls of both sexes. Note: Weight was only assessed directly after behavioral testing, before LABORAS, or at weaning to avoid additionally disrupting animals during resting periods between tests. Sample sizes vary due to the following reasons: (1) Weight after measuring USVs at P8, P12, and P16 was not initially measured in the first two litters and was later added as an additional outcome to analyze weight differences before weaning. (2) Animal numbers were reduced after P26 due to capacity limits (e.g., LABORAS system availability, feasibility of long behavioral protocols like the three-chamber test). Animal selection for further testing was done by an unbiased person who was only aware of genotypes and cage distributions. This ensured a balanced genotype distribution while maintaining practical feasibility in reducing sample size. The experimenter remained blind to the genotypes. (3) The Spontaneous Alternation T-Maze was performed on P37-38. Animals tested and weighed on P38 were excluded from weight analysis in this case. Especially considering the robust genotype effects observed across all time points, we have no reason to believe that the varying sample sizes introduced any bias into the results.

Figure	Parameter	Statistical test	Source of variation	P Value	F (DFn, DFd)
Fig. 1c	P8 Weight	two-way ANOVA	Genotype	**<0.0001********	F (1, 43) = 18.92
			Sex	**0.0180***	F (1, 43) = 6.055
			Genotype x sex	0.1931	F (1, 43) = 1.748
Fig. 1c	P12 Weight	two-way ANOVA	Genotype	**0.0002*****	F (1, 43) = 16.690
			Sex	**0.0120***	F (1, 43) = 6.887
			Genotype x sex	0.1331	F (1, 43) = 2.343
Fig. 1c	P16 Weight	two-way ANOVA	Genotype	**0.0003*****	F (1, 43) = 15.810
			Sex	**0.0193***	F (1, 43) = 5.906
			Genotype x sex	0.1616	F (1, 43) = 2.028
Fig. 1c	P21 Weight	two-way ANOVA	Genotype	**<0.0001********	F (1, 69) = 30.730
			Sex	**0.0085****	F (1, 69) = 7.346
			Genotype x sex	0.1656	F (1, 69) = 1.936
Fig. 1c	P26 Weight	two-way ANOVA	Genotype	**<0.0001********	F (1, 66) = 19.670
			Sex	**0.0061****	F (1, 66) = 8.024
			Genotype x sex	0.4327	F (1, 66) = 0.6232
Fig. 1c	P35 Weight	two-way ANOVA	Genotype	**<0.0001********	F (1, 55) = 34.960
			Sex	**<0.0001********	F (1, 55) = 34.060
			Genotype x sex	0.1238	F (1, 55) = 2.443
Fig. 1c	P37 Weight	two-way ANOVA	Genotype	**<0.0001********	F (1, 50) = 24.630
			Sex	**<0.0001********	F (1, 50) = 38.730
			Genotype x sex	0.2881	F (1, 50) = 1.153

Supplementary Table S2. Statistical comparisons of body weight at different time points for effects of genotype, sex, and genotype-by-sex interaction in *Magel2*^{Pmut} rats and wild-type controls (two-way ANOVA).

Figure	Parameter	Sample size before outlier analysis				Outliers removed (ROUT, Q = 1%)	Sample size after outlier analysis			
		WT male	WT female	Pmut male	Pmut female		WT male	WT female	Pmut male	Pmut female
Fig. 2b	P8 USVs Call length	20	14	16	24	1	20	14	15	24
Fig. 2b	P12 USVs Call length	20	14	16	24	0	20	14	16	24
Fig. 2b	P16 USVs Call length	20	14	16	24	1	20	14	15	24
Fig. 2c	P8 USVs Sinuosity	20	14	16	24	1	19	14	16	24
Fig. 2c	P12 USVs Sinuosity	20	14	16	24	1	20	14	16	23
Fig. 2c	P16 USVs Sinuosity	20	14	16	24	0	20	14	16	24
Fig. 2d	P8 USVs Power	20	14	16	24	0	20	14	16	24
Fig. 2d	P12 USVs Power	20	14	16	24	1	19	14	16	24
Fig. 2d	P16 USVs Power	20	14	16	24	0	20	14	16	24
Fig. S2	P8 USVs Number of calls	20	14	16	24	0	20	14	16	24
Fig. S2	P12 USVs Number of calls	20	14	16	24	0	20	14	16	24
Fig. S2	P16 USVs Number of calls	20	14	16	24	1	19	14	16	24
Fig. S2	P8 USVs Prin. frequency	20	14	16	24	0	20	14	16	24
Fig. S2	P12 USVs Prin. frequency	20	14	16	24	0	20	14	16	24
Fig. S2	P16 USVs Prin. frequency	20	14	16	24	0	20	14	16	24
Fig. S2	P8 USVs Min. frequency	20	14	16	24	0	20	14	16	24
Fig. S2	P12 USVs Min. frequency	20	14	16	24	0	20	14	16	24
Fig. S2	P16 USVs Min. frequency	20	14	16	24	0	20	14	16	24
Fig. S2	P8 USVs Max. frequency	20	14	16	24	0	20	14	16	24
Fig. S2	P12 USVs Max. frequency	20	14	16	24	0	20	14	16	24
Fig. S2	P16 USVs Max. frequency	20	14	16	24	0	20	14	16	24
Fig. S2	P8 USVs Tonality	20	14	16	24	0	20	14	16	24
Fig. S2	P12 USVs Tonality	20	14	16	24	1	19	14	16	24
Fig. S2	P16 USVs Tonality	20	14	16	24	3	20	14	15	22

Supplementary Table S3. Sample size before and after outlier analysis (ROUT, Q = 1%) for isolation-induced ultrasonic vocalization (USV) parameters in *Magel2*^{Pmut} rats and wild-type controls of both sexes. Prin.: Principal; Min.: Minimum; Max.: Maximum.

Figure	Parameter	Statistical test	Source of variation	P Value	F (DFn, DFd)
Fig. 2b	P8 USVs<br>Call length	two-way ANOVA	Genotype	**0.0001*** **	F (1, 69) = 17.00
			Sex	**0.0132***	F (1, 69) = 6.468
			Genotype x sex	0.3186	F (1, 69) = 1.009
Fig. 2b	P12 USVs<br>Call length	two-way ANOVA	Genotype	**0.0298***	F (1, 70) = 4.922
			Sex	0.1596	F (1, 70) = 2.021
			Genotype x sex	**0.0030****	F (1, 70) = 9.435
Fig. 2b	P16 USVs<br>Call length	two-way ANOVA	Genotype	0.7252	F (1, 69) = 0.1246
			Sex	0.0622	F (1, 69) = 3.592
			Genotype x sex	**0.0041****	F (1, 69) = 8.807
Fig. 2c	P8 USVs<br>Sinuosity	two-way ANOVA	Genotype	0.9216	F (1, 69) = 0.009748
			Sex	0.2279	F (1, 69) = 1.480
			Genotype x sex	0.4139	F (1, 69) = 0.6757
Fig. 2c	P12 USVs<br>Sinuosity	two-way ANOVA	Genotype	0.7504	F (1, 69) = 0.1020
			Sex	0.1603	F (1, 69) = 2.015
			Genotype x sex	**0.0010*****	F (1, 69) = 11.88
Fig. 2c	P16 USVs<br>Sinuosity	two-way ANOVA	Genotype	0.0832	F (1, 70) = 3.089
			Sex	0.2045	F (1, 70) = 1.641
			Genotype x sex	0.0698	F (1, 70) = 3.390
Fig. 2d	P8 USVs<br>Power	two-way ANOVA	Genotype	0.1244	F (1, 70) = 0.1244
			Sex	0.1738	F (1, 70) = 0.1738
			Genotype x sex	0.6507	F (1, 70) = 0.6507
Fig. 2d	P12 USVs<br>Power	two-way ANOVA	Genotype	**0.0107***	F (1, 69) = 6.893
			Sex	**0.0320***	F (1, 69) = 4.793
			Genotype x sex	0.3254	F (1, 69) = 0.9812
Fig. 2d	P16 USVs<br>Power	two-way ANOVA	Genotype	0.7109	F (1, 70) = 0.1385
			Sex	0.7724	F (1, 70) = 0.08430
			Genotype x sex	0.2225	F (1, 70) = 1.515
Fig. S2	P8 USVs<br>Number of calls	two-way ANOVA	Genotype	0.4533	F (1, 70) = 0.5687
			Sex	0.9595	F (1, 70) = 0.002598
			Genotype x sex	0.7667	F (1, 70) = 0.08869
Fig. S2	P12 USVs<br>Number of calls	two-way ANOVA	Genotype	0.4827	F (1, 70) = 0.4980
			Sex	0.8650	F (1, 70) = 0.02912
			Genotype x sex	**0.0236***	F (1, 70) = 5.354
Fig. S2	P16 USVs<br>Number of calls	two-way ANOVA	Genotype	0.2081	F (1, 69) = 1.614
			Sex	0.5979	F (1, 69) = 0.2807
			Genotype x sex	0.7148	F (1, 69) = 0.1346
Fig. S2	P8 USVs<br>Prin. frequency	two-way ANOVA	Genotype	0.3316	F (1, 70) = 0.9557
			Sex	0.2201	F (1, 70) = 1.531
			Genotype x sex	0.3321	F (1, 70) = 0.9539
Fig. S2	P12 USVs<br>Prin. frequency	two-way ANOVA	Genotype	0.7190	F (1, 70) = 0.1305
			Sex	0.0824	F (1, 70) = 3.105
			Genotype x sex	0.1000	F (1, 70) = 2.779
Fig. S2	P16 USVs<br>Prin. frequency	two-way ANOVA	Genotype	0.9676	F (1, 70) = 0.001664
			Sex	**0.0399***	F (1, 70) = 4.383
			Genotype x sex	**0.0304***	F (1, 70) = 4,880
Fig. S2	P8 USVs<br>Min. frequency	two-way ANOVA	Genotype	0.2801	F (1, 70) = 1.185
			Sex	0.1255	F (1, 70) = 2.405
			Genotype x sex	0.4330	F (1, 70) = 0.6219
Fig. S2	P12 USVs<br>Min. frequency	two-way ANOVA	Genotype	0.3249	F (1, 70) = 0.9829
			Sex	0.0863	F (1, 70) = 3.026
			Genotype x sex	0.2157	F (1, 70) = 1.561
Fig. S2	P16 USVs<br>Min. frequency	two-way ANOVA	Genotype	0.8694	F (1, 70) = 0.02722
			Sex	**0.0499***	F (1, 70) = 3.980
			Genotype x sex	**0.0321***	F (1, 70) = 4.784
Fig. S2	P8 USVs<br>Max. frequency	two-way ANOVA	Genotype	0.2744	F (1, 70) = 1.213
			Sex	0.0858	F (1, 70) = 3.036
			Genotype x sex	0.7496	F (1, 70) = 0.1027

Fig. S2	P12 USVs Max. frequency	two-way ANOVA	Genotype	0.6764	F (1, 70) = 0.1757
			Sex	0.0519	F (1, 70) = 3.913
			Genotype x sex	**0.0126***	F (1, 70) = 6.556
Fig. S2	P16 USVs Max. frequency	two-way ANOVA	Genotype	0.9182	F (1, 70) = 0.01062
			Sex	0.1149	F (1, 70) = 2.549
			Genotype x sex	**0.0186***	F (1, 70) = 5.804
Fig. S2	P8 USVs Tonality	two-way ANOVA	Genotype	0.0794	F (1, 70) = 3.168
			Sex	0.0845	F (1, 70) = 3.063
			Genotype x sex	0.8427	F (1, 70) = 0.03965
Fig. S2	P12 USVs Tonality	two-way ANOVA	Genotype	**0.0032****	F (1, 69) = 9.345
			Sex	0.0674	F (1, 69) = 3.454
			Genotype x sex	0.2599	F (1, 69) = 1.290
Fig. S2	P16 USVs Tonality	two-way ANOVA	Genotype	**0.0474***	F (1, 67) = 4.080
			Sex	0.8689	F (1, 67) = 0.02746
			Genotype x sex	0.1915	F (1, 67) = 1.741

Supplementary Table S4. Statistical comparisons of isolation-induced ultrasonic vocalization (USV) parameters for effects of genotype, sex, and genotype-by-sex interaction in *Magel2*^{Pmut} rats and wild-type controls (two-way ANOVA). Prin.: Principal; Min.: Minimum; Max.: Maximum.

**Figure**	**Parameter**	**Statistical test**	**Comparison**	**Adjusted P Value**	**t**
Fig. 2b	P12 USVs Call length	*post hoc* t-test with Bonferroni correction	WT vs. Pmut – Male	**0.0007*****	3.746
			WT vs. Pmut – Female	>0.9999	0.6024
Fig. 2b	P16 USVs Call length	*post hoc* t-test with Bonferroni correction	WT vs. Pmut – Male	**0.0455***	2.330
			WT vs. Pmut – Female	0.1333	1.863
Fig. 2c	P12 USVs Sinuosity	*post hoc* t-test with Bonferroni correction	WT vs. Pmut – Male	**0.0185***	2.677
			WT vs. Pmut – Female	0.0624	2.200
Fig. S2	P12 USVs Number of calls	*post hoc* t-test with Bonferroni correction	WT vs. Pmut – Male	0.5175	1.139
			WT vs. Pmut – Female	0.0730	2.132
Fig. S2	P16 USVs Prin. frequency	*post hoc* t-test with Bonferroni correction	WT vs. Pmut – Male	0.2313	1.593
			WT vs. Pmut – Female	0.2605	1.531
Fig. S2	P16 USVs Min. frequency	*post hoc* t-test with Bonferroni correction	WT vs. Pmut – Male	0.3133	1.432
			WT vs. Pmut – Female	0.2023	1.661
Fig. S2	P12 USVs Max. frequency	*post hoc* t-test with Bonferroni correction	WT vs. Pmut – Male	0.0769	2.110
			WT vs. Pmut – Female	0.2700	1.512
Fig. S2	P16 USVs Max. frequency	*post hoc* t-test with Bonferroni correction	WT vs. Pmut – Male	0.1592	1.779
			WT vs. Pmut – Female	0.2158	1.629

Supplementary Table S5. Sex-specific *post hoc* comparisons following detection of a significant genotype-by-sex interaction in isolation-induced ultrasonic vocalization (USV) parameters in *Magel2*^{Pmut} rats and wild-type controls (*post hoc* unpaired, two-tailed t-tests with Bonferroni correction). Prin.: Principal; Min.: Minimum; Max.: Maximum.

Figure	Parameter	Sample size before outlier analysis				Outliers removed (ROUT, Q = 1%)	Sample size after outlier analysis			
		WT male	WT female	Pmut male	Pmut female		WT male	WT female	Pmut male	Pmut female
Fig. 3a	EPM - Time in open arms	20	14	16	24	1	19	14	16	24
Fig. 3b	EPM – Distance in open arms	20	14	16	24	3	18	13	16	24
Fig. 3c	EPM - Number of open arms visits	20	14	16	24	0	20	14	16	24
Fig. 3d	EPM - Time in closed arms	20	14	16	24	0	20	14	16	24

Supplementary Table S6. Sample size before and after outlier analysis (ROUT, Q = 1%) for elevated plus maze (EPM) parameters in *Magel2*^{Pmut} rats and wild-type controls of both sexes.

Figure	Parameter	Statistical test	Source of variation	P Value	F (DFn, DFd)
Fig. 3a	EPM - Time in open arms	two-way ANOVA	Genotype	**0.0048****	F (1, 69) = 8.475
			Sex	0.5103	F (1, 69) = 0.4380
			Genotype x sex	0.8510	F (1, 69) = 0.03555
Fig. 3b	EPM - Distance in open arms	two-way ANOVA	Genotype	**0.0013****	F (1, 67) = 11.20
			Sex	0.6239	F (1, 67) = 0.2427
			Genotype x sex	0.8536	F (1, 67) = 0.03430
Fig. 3c	EPM – Number of open arms visits	two-way ANOVA	Genotype	**0.0230***	F (1, 70) = 5.402
			Sex	0.8750	F (1, 70) = 0.02494
			Genotype x sex	0.2682	F (1, 70) = 1.246
Fig. 3d	EPM - Time in closed arms	two-way ANOVA	Genotype	**0.0443***	F (1, 70) = 4.195
			Sex	0.6699	F (1, 70) = 0.1833
			Genotype x sex	0.7244	F (1, 70) = 0.1253

Supplementary Table S7. Statistical comparisons of elevated plus maze (EPM) parameters for effects of genotype, sex, and genotype-by-sex interaction in *Magel2*^{Pmut} rats and wild-type controls (two-way ANOVA).

Figure	Parameter	Sample size before outlier analysis				Outliers removed (ROUT, Q = 1%)	Sample size after outlier analysis			
		WT male	WT female	Pmut male	Pmut female		WT male	WT female	Pmut male	Pmut female
Fig. S3	OF – Total distance traveled	20	14	16	24	0	20	14	16	24
Fig. S3	OF – Time in center	20	14	16	24	6	19	13	13	23
Fig. S3	OF – Distance in center	20	14	16	24	4	20	13	13	24
Fig. S3	OF – Time in edges	20	14	16	24	0	20	14	16	24

Supplementary Table S8. Sample size before and after outlier analysis (ROUT, Q = 1%) for open field (OF) parameters in *Magel2*^{Pmut} rats and wild-type controls of both sexes.

Figure	Parameter	Statistical test	Source of variation	P Value	F (DFn, DFd)
Fig. S3	OF – Total distance traveled	two-way ANOVA	Genotype	0.2722	F (1, 70) = 1.225
			Sex	0.5387	F (1, 70) = 0.3817
			Genotype x sex	0.7319	F (1, 70) = 0.1183
Fig. S3	OF – Time in center	two-way ANOVA	Genotype	0.5711	F (1, 64) = 0.3242
			Sex	0.2632	F (1, 64) = 1.274
			Genotype x sex	0.3934	F (1, 64) = 0.7384
Fig. S3	OF – Distance in center	two-way ANOVA	Genotype	0.9355	F (1, 66) = 0.006601
			Sex	0.8235	F (1, 66) = 0.05016
			Genotype x sex	0.7032	F (1, 66) = 0.1465
Fig. S3	OF – Time in edges	two-way ANOVA	Genotype	0.6418	F (1, 70) = 0.2183
			Sex	0.3569	F (1, 70) = 0.8601
			Genotype x sex	0.1832	F (1, 70) = 1.807

Supplementary Table S9. Statistical comparisons of open field (OF) parameters for effects of genotype, sex, and genotype-by-sex interaction in *Magel2*^{Pmut} rats and wild-type controls (two-way ANOVA).

Figure	Parameter	Sample size before outlier analysis				Outliers removed (ROUT, Q = 1%)	Sample size after outlier analysis			
		WT male	WT female	Pmut male	Pmut female		WT male	WT female	Pmut male	Pmut female
Fig. 4b	LABORAS Eating duration	16	13	14	16	1	16	13	13	16
Fig. 4c	LABORAS Drinking duration	16	13	14	16	0	16	13	14	16
Fig. 4d	LABORAS Eating events	16	13	14	16	0	16	13	14	16
Fig. 4e	LABORAS Drinking events	16	13	14	16	0	16	13	14	16
Fig. 4f	LABORAS Weight change	16	13	14	16	3	16	13	12	15
Fig. 4g	LABORAS Locom. duration	16	13	14	16	0	16	13	14	16
Fig. 4h	LABORAS Immobil. duration	16	13	14	16	0	16	13	14	16
Fig. 4i	LABORAS Groom. duration	16	13	14	16	0	16	13	14	16
Fig. S4	LABORAS Locom. events	16	13	14	16	0	16	13	14	16
Fig. S4	LABORAS Immobil. events	16	13	14	16	0	16	13	14	16
Fig. S4	LABORAS Groom. events	16	13	14	16	0	16	13	14	16
Fig. S4	LABORAS Distance traveled	16	13	14	16	0	16	13	14	16

Supplementary Table S10. Sample size before and after outlier analysis (ROUT, Q = 1%) for LABORAS home-cage monitoring parameters in *Magel2*^{Pmut} rats and wild-type controls of both sexes. Note: One female *Magel2*^{Pmut} animal was excluded from LABORAS due to not reaching the minimum required weight of 80g in time. Locom.: Locomotion; Immobil.: Immobility; Groom: Grooming.

Figure	Parameter	Statistical test	Source of variation	P Value	F (DFn, DFd)
Fig. 4b	LABORAS Eating duration	two-way ANOVA	Genotype	**0.0002*** **	F (1, 54) = 16.54
			Sex	0.5679	F (1, 54) = 0.1609
			Genotype x sex	0.1609	F (1, 54) = 2.021
Fig. 4c	LABORAS Drinking duration	two-way ANOVA	Genotype	**0.0301***	F (1, 55) = 4.958
			Sex	0.3838	F (1, 55) = 0.7707
			Genotype x sex	0.8078	F (1, 55) = 0.05976
Fig. 4d	LABORAS Eating events	two-way ANOVA	Genotype	**0.0099****	F (1, 55) = 7.141
			Sex	0.6256	F (1, 55) = 0.2408
			Genotype x sex	0.5268	F (1, 55) = 0.4057
Fig. 4e	LABORAS Drinking events	two-way ANOVA	Genotype	**0.0187***	F (1, 55) = 5.869
			Sex	0.3853	F (1, 55) = 0.7659
			Genotype x sex	0.9077	F (1, 55) = 0.01357
Fig. 4f	LABORAS Weight change	two-way ANOVA	Genotype	0.2127	F (1, 52) = 1.592
			Sex	0.2387	F (1, 52) = 1.421
			Genotype x sex	0.3785	F (1, 52) = 0.7888
Fig. 4g	LABORAS Locomotion duration	two-way ANOVA	Genotype	0.3344	F (1, 55) = 0.9485
			Sex	0.0739	F (1, 55) = 3.319
			Genotype x sex	0.3008	F (1, 55) = 1.091
Fig. 4h	LABORAS Immobility duration	two-way ANOVA	Genotype	0.2944	F (1, 55) = 1.121
			Sex	0.1574	F (1, 55) = 2.055
			Genotype x sex	0.6963	F (1, 55) = 0.1540
Fig. 4i	LABORAS Grooming duration	two-way ANOVA	Genotype	0.2811	F (1, 55) = 1.185
			Sex	0.3520	F (1, 55) = 0.8812
			Genotype x sex	0.8311	F (1, 55) = 0.04591
Fig. S4	LABORAS Locomotion events	two-way ANOVA	Genotype	0.5807	F (1, 55) = 0.3088
			Sex	0.1597	F (1, 55) = 2.032
			Genotype x sex	0.3004	F (1, 55) = 1.093
Fig. S4	LABORAS Immobility events	two-way ANOVA	Genotype	0.2734	F (1, 55) = 1.224
			Sex	0.9736	F (1, 55) = 0.001106
			Genotype x sex	0.4746	F (1, 55) = 0.5183
Fig. S4	LABORAS Grooming evens	two-way ANOVA	Genotype	0.1779	F (1, 55) = 1.862
			Sex	**0.0483***	F (1, 55) = 4.079
			Genotype x sex	0.3309	F (1, 55) = 0.9624
Fig. S4	LABORAS Distance traveled	two-way ANOVA	Genotype	0.1170	F (1, 55) = 2.536
			Sex	0.5736	F (1, 55) = 0.3204
			Genotype x sex	0.5816	F (1, 55) = 0.3072

Supplementary Table S11. Statistical comparisons of LABORAS home-cage monitoring parameters for effects of genotype, sex, and genotype-by-sex interaction in *Magel2*^{Pmut} rats and wild-type controls (two-way ANOVA).

Figure	Parameter	Sample size before outlier analysis				Outliers removed (ROUT, Q = 1%)	Sample size after outlier analysis			
		WT male	WT female	Pmut male	Pmut female		WT male	WT female	Pmut male	Pmut female
Fig. 5c	CatWalk XT Run avg. speed	16	13	14	17	0	16	13	14	17
Fig. 5d	CatWalk XT Number of steps	16	13	14	17	1	16	13	13	17
Fig. 5e	CatWalk XT Num. of patterns	16	13	14	17	0	16	13	14	17
Fig. 5f	CatWalk XT Stride length	16	13	14	17	0	16	13	14	17
Fig. 5g	CatWalk XT Paw print area	16	13	14	17	1	16	13	14	16
Fig. 5h	CatWalk XT Swing speed	16	13	14	17	0	16	13	14	17
Fig. 5i	CatWalk XT Hind paw width	16	13	14	17	0	16	13	14	17
Fig. 5j	CatWalk XT Regularity index	16	13	14	17	0	16	13	14	17
Fig. S5	CatWalk XT Support single	16	13	14	17	5	14	13	13	15
Fig. S5	CatWalk XT Support diagonal	16	13	14	17	0	16	13	14	17
Fig. S5	CatWalk XT Support lateral	16	13	14	17	0	16	13	14	17
Fig. S5	CatWalk XT Support three	16	13	14	17	0	16	13	14	17
Fig. S5	CatWalk XT Support four	16	13	14	17	3	16	12	13	16
Fig. S5	CatWalk XT Run duration	16	13	14	17	1	16	13	13	17
Fig. S5	CatWalk XT Paw print width	16	13	14	17	0	16	13	14	17
Fig. S5	CatWalk XT Paw print length	16	13	14	17	2	16	12	14	16
Fig. S5	CatWalk XT Swing time	16	13	14	17	0	16	13	14	17
Fig. S5	CatWalk XT Stand time	16	13	14	17	1	16	12	13	17
Fig. S5	CatWalk XT Cadence	16	13	14	17	0	16	13	14	17
Fig. S5	CatWalk XT Front paw width	16	13	14	17	0	16	13	14	17

Supplementary Table S12. Sample size before and after outlier analysis (ROUT, Q = 1%) for CatWalk XT parameters in *Magel2*^{Pmut} rats and wild-type controls of both sexes. avg.: average.

Figure	Parameter	Statistical test	Source of variation	P Value	F (DFn, DFd)
Fig. 5c	CatWalk XT Run avg. speed	two-way ANOVA	Genotype	0.3584	F (1, 56) = 0.8575
			Sex	0.2248	F (1, 56) = 1.507
			Genotype x sex	0.3219	F (1, 56) = 0.9990
Fig. 5d	CatWalk XT Number of steps	two-way ANOVA	Genotype	**0.0041****	F (1, 55) = 8.989
			Sex	0.8965	F (1, 55) = 0.01708
			Genotype x sex	0.0805	F (1, 55) = 3.172
Fig. 5e	CatWalk XT Num. of patterns	two-way ANOVA	Genotype	**0.0091****	F (1, 56) = 7.297
			Sex	0.5618	F (1, 56) = 0.3406
			Genotype x sex	0.1353	F (1, 56) = 2.296
Fig. 5f	CatWalk XT Stride length	two-way ANOVA	Genotype	**0.0038****	F (1, 56) = 9.125
			Sex	0.6128	F (1, 56) = 0.2591
			Genotype x sex	0.0640	F (1, 56) = 3.569
Fig. 5g	CatWalk XT Paw print area	two-way ANOVA	Genotype	**<0.0001******	F (1, 55) = 22.22
			Sex	**0.003*****	F (1, 55) = 14.76
			Genotype x sex	0.7426	F (1, 55) = 0.7426
Fig. 5h	CatWalk XT Swing speed	two-way ANOVA	Genotype	**0.0342***	F (1, 56) = 4.715
			Sex	0.7782	F (1, 56) = 0.08009
			Genotype x sex	0.3601	F (1, 56) = 0.8515
Fig. 5i	CatWalk XT Hind paw width	two-way ANOVA	Genotype	**0.0025****	F (1, 56) = 10.02
			Sex	**0.0086****	F (1, 56) = 7.414
			Genotype x sex	**0.0102***	F (1, 56) = 7.061
Fig. 5i	CatWalk XT Regularity index	two-way ANOVA	Genotype	**0.0069****	F (1, 56) = 7.876
			Sex	0.5710	F (1, 56) = 0.3249
			Genotype x sex	**0.0278***	F (1, 56) = 5.100
Fig. S5	CatWalk XT Support single	two-way ANOVA	Genotype	0.4015	F (1, 51) = 0.7157
			Sex	**0.0031****	F (1, 51) = 9.609
			Genotype x sex	0.4681	F (1, 51) = 0.5345
Fig. S5	CatWalk XT Support diagonal	two-way ANOVA	Genotype	0.9091	F (1, 56) = 0.01315
			Sex	0.3919	F (1, 56) = 0.7445
			Genotype x sex	0.2360	F (1, 56) = 1.435
Fig. S5	CatWalk XT Support lateral	two-way ANOVA	Genotype	0.5969	F (1, 56) = 0.2830
			Sex	**0.0187***	F (1, 56) = 5.863
			Genotype x sex	0.1380	F (1, 56) = 2.264
Fig. S5	CatWalk XT Support three	two-way ANOVA	Genotype	0.7085	F (1, 56) = 0.1413
			Sex	0.2243	F (1, 56) = 1.510
			Genotype x sex	0.2863	F (1, 56) = 1.159
Fig. S5	CatWalk XT Support four	two-way ANOVA	Genotype	0.0744	F (1, 53) = 3.312
			Sex	0.0887	F (1, 53) = 3.007
			Genotype x sex	0.4816	F (1, 53) = 0.5022
Fig. S5	CatWalk XT Run duration	two-way ANOVA	Genotype	0.4043	F (1, 55) = 0.7064
			Sex	0.0966	F (1, 55) = 2.857
			Genotype x sex	0.7813	F (1, 55) = 0.07785
Fig. S5	CatWalk XT Paw print width	two-way ANOVA	Genotype	**<0.0001******	F (1, 56) = 21.61
			Sex	**0.0011****	F (1, 56) = 11.83
			Genotype x sex	0.9152	F (1, 56) = 0.01144
Fig. S5	CatWalk XT Paw print length	two-way ANOVA	Genotype	**0.0001*****	F (1, 54) = 17.27
			Sex	**0.0016****	F (1, 54) = 11.05
			Genotype x sex	0.5205	F (1, 54) = 0.4183
Fig. S5	CatWalk XT Swing time	two-way ANOVA	Genotype	0.3269	F (1, 56) = 0.9782
			Sex	0.3987	F (1, 56) = 0.7231
			Genotype x sex	0.4893	F (1, 56) = 0.4845
Fig. S5	CatWalk XT Stand time	two-way ANOVA	Genotype	0.7564	F (1, 55) = 0.09724
			Sex	0.0666	F (1, 55) = 3.501
			Genotype x sex	0.2880	F (1, 55) = 1.151
Fig. S5	CatWalk XT Cadence	two-way ANOVA	Genotype	0.8058	F (1, 56) = 0.06099
			Sex	0.2923	F (1, 56) = 1.130
			Genotype x sex	0.2388	F (1, 56) = 1.418

Fig. S5	CatWalk XT Front paw width	two-way ANOVA	Genotype	0.6111	F (1, 56) = 0.2615
			Sex	0.5691	F (1, 56) = 0.3281
			Genotype x sex	0.8609	F (1, 56) = 0.03100

Supplementary Table S13. Statistical comparisons of CatWalk XT parameters for effects of genotype, sex, and genotype-by-sex interaction in *Magel2*^{Pmut} rats and wild-type controls (two-way ANOVA). avg.: average.

Figure	Parameter	Statistical test	Comparison	Adjusted P Value	t
Fig. 5i	CatWalkXT Regularity index	*post hoc* t-test with Bonferroni correction	WT vs. Pmut – Male	**0.0014****	3.593
			WT vs. Pmut – Female	>0.9999	0.3863
Fig. 5j	CatWalkXT BOS hind paws	*post hoc* t-test with Bonferroni correction	WT vs. Pmut – Male	>0.9999	0.3601
			WT vs. Pmut – Female	**0.0003*****	4.103

Supplementary Table S14. Sex-specific *post hoc* comparisons following detection of a significant genotype-by-sex interaction in CatWalk XT parameters in *Magel2*^{Pmut} rats and wild-type controls (*post hoc* unpaired, two-tailed t-tests with Bonferroni correction).

Figure	Parameter	Sample size before outlier analysis				Outliers removed (ROUT, Q = 1%)	Sample size after outlier analysis			
		WT male	WT female	Pmut male	Pmut female		WT male	WT female	Pmut male	Pmut female
Fig. 6a	T-maze Alternation rate	16	13	14	17	0	16	13	14	17
Fig. 6b	T-maze Completion rate	16	13	14	17	1	16	12	14	17
Fig. 6c	T-maze Decision time	16	13	14	17	0	16	13	14	17

Supplementary Table S15. Sample size before and after outlier analysis (ROUT, Q = 1%) for spontaneous alternation T-maze parameters in *Magel2*^{Pmut} rats and wild-type controls of both sexes.

Figure	Parameter	Statistical test	Source of variation	P Value	F (DFn, DFd)
Fig. 6a	T-maze Alternation rate	two-way ANOVA	Genotype	0.6150	F (1, 56) = 0.2559
			Sex	0.4715	F (1, 56) = 0.5256
			Genotype x sex	0.4051	F (1, 56) = 0.7037
Fig. 6b	T-maze Completion rate	two-way ANOVA	Genotype	0.6438	F (1, 55) = 0.2162
			Sex	0.1829	F (1, 55) = 1.819
			Genotype x sex	0.3319	F (1, 55) = 0.3319
Fig. 6c	T-maze Decision time	two-way ANOVA	Genotype	**0.0063****	F (1, 56) = 8.055
			Sex	0.8710	F (1, 56) = 0.02661
			Genotype x sex	0.9464	F (1, 56) = 0.004564

Supplementary Table S16. Statistical comparisons of spontaneous alternation T-maze parameters for effects of genotype, sex, and genotype-by-sex interaction in *Magel2*^{Pmut} rats and wild-type controls (two-way ANOVA).

Figure	Parameter	Sample size before outlier analysis				Outliers removed (ROUT, Q = 1%)	Sample size after outlier analysis			
		WT male	WT female	Pmut male	Pmut female		WT male	WT female	Pmut male	Pmut female
Fig. S6	TCT – Sociability Time in prox. of S1	16	13	14	17	0	16	13	14	17
Fig. S6	TCT – Sociability Time in prox. of E	16	13	14	17	1	16	12	14	17
Fig. S6	TCT – Sociability Sociability index	16	13	14	17	2	15	13	14	16
Fig. S6	TCT – Soc. novelty Time in prox. of S1	16	13	14	17	0	16	13	14	17
Fig. S6	TCT – Soc. novelty Time in prox. of S2	16	13	14	17	0	16	13	14	17
Fig. S6	TCT – Soc. novelty Soc. novelty index	16	13	14	17	0	16	13	14	17

Supplementary Table S17. Sample size before and after outlier analysis (ROUT, Q = 1%) for three-chamber social interaction test (TCT) parameters in *Magel2*^{Pmut} rats and wild-type controls of both sexes. S1: Stranger 1; E: Empty cup; S2: Stranger 2.

Figure	Parameter	Statistical test	Source of variation	P Value	F (DFn, DFd)
Fig. S6	TCT Sociability: Time in prox. of S1	two-way ANOVA	Genotype	0.7611	F (1, 56) = 0.09333
			Sex	0.2513	F (1, 56) = 1.344
			Genotype x sex	0.1142	F (1, 56) = 2.575
Fig. S6	TCT Sociability: Time in prox. of E	two-way ANOVA	Genotype	0.7722	F (1, 55) = 0.08465
			Sex	0.1133	F (1, 55) = 2.589
			Genotype x sex	0.7757	F (1, 55) = 0.08202
Fig. S6	TCT – Sociability: Sociability index	two-way ANOVA	Genotype	0.2752	F (1, 54) = 1.215
			Sex	0.3963	F (1, 54) = 0.7312
			Genotype x sex	0.2564	F (1, 54) = 1.316
Fig. S6	TCT Soc. nov.: Time in prox. of S1	two-way ANOVA	Genotype	0.2805	F (1, 56) = 1.188
			Sex	0.9279	F (1, 56) = 0.008258
			Genotype x sex	0.5845	F (1, 56) = 0.3025
Fig. S6	TCT Soc. nov.: Time in prox. of S2	two-way ANOVA	Genotype	0.9920	F (1, 56) = 0.000102
			Sex	0.3988	F (1, 56) = 0.7230
			Genotype x sex	0.3000	F (1, 56) = 1.095
Fig. S6	TCT Soc. nov.: Soc. nov. index	two-way ANOVA	Genotype	0.9785	F (1, 56) = 0.000734
			Sex	0.4352	F (1, 56) = 0.6177
			Genotype x sex	0.4513	F (1, 56) = 0.5754

Supplementary Table S18. Statistical comparisons of the three-chamber social interaction test (TCT) parameters for effects of genotype, sex, and genotype-by-sex interaction in *Magel2*^{Pmut} rats and wild-type controls (two-way ANOVA). S1: Stranger 1; E: Empty cup; S2: Stranger 2.

Figure	Parameter	Sample size before outlier analysis				Outliers removed (ROUT, Q = 1%)	Sample size after outlier analysis			
		WT male	WT female	Pmut male	Pmut female		WT male	WT female	Pmut male	Pmut female
Fig. 7b	DSI – Social behaviors duration	14	13	14	17	0	14	13	14	17
Fig. 7c	DSI – Non-social behaviors duration	14	13	14	17	1	14	13	14	16
Fig. 7d	DSI – Crawling duration	14	13	14	17	1	14	13	13	17
Fig. 7e	DSI – Body sniffing duration	14	13	14	17	0	14	13	14	17
Fig. 7f	DSI – Nose-to-nose cont. duration	14	13	14	17	3	14	12	12	17
Fig. 7g	DSI – Rearing duration	14	13	14	17	5	12	12	13	16
Fig. 7h	DSI – Play behaviors duration	14	13	14	17	0	14	13	14	17
Fig. 7i	DSI – Following duration	14	13	14	17	0	14	13	14	17
Fig. 7j	DSI – Anogenital sniffing duration	14	13	14	17	0	14	13	14	16
Fig. 7k	DSI – Self-grooming duration	14	13	14	17	1	14	13	14	16

Supplementary Table S19. Sample size before and after outlier analysis (ROUT, Q = 1%) for direct social interaction (DSI) test parameters in *Magel2*^{Pmut} rats and wild-type controls of both sexes. Note: Two male *Magel2*^{Pmut} animals were excluded from video analysis due to technical problems.

Figure	Parameter	Statistical test	Source of variation	P Value	F (DFn, DFd)
Fig. 7b	DSI – Social behaviors duration	two-way ANOVA	Genotype	0.0987	F (1, 54) = 2.823
			Sex	0.4783	F (1, 54) = 0.5098
			Genotype x sex	0.8478	F (1, 54) = 0.03721
Fig. 7c	DSI – Non-social behaviors duration	two-way ANOVA	Genotype	0.1506	F (1, 53) = 2.127
			Sex	0.6645	F (1, 53) = 0.1902
			Genotype x sex	0.5058	F (1, 53) = 0.4489
Fig. 7d	DSI – Crawling duration	two-way ANOVA	Genotype	**<0.0001******	F (1, 53) = 18.46
			Sex	**0.0117***	F (1, 53) = 6.823
			Genotype x sex	0.4991	F (1, 53) = 0.4633
Fig. 7e	DSI – Body sniffing duration	two-way ANOVA	Genotype	**0.0473***	F (1, 54) = 0.0473
			Sex	0.5671	F (1, 54) = 0.5671
			Genotype x sex	0.1062	F (1, 54) = 0.1062
Fig. 7f	DSI – Nose-to-nose contact duration	two-way ANOVA	Genotype	**0.0004*****	F (1, 51) = 14.49
			Sex	**0.0005*****	F (1, 51) = 14.02
			Genotype x sex	**0.0008*****	F (1, 51) = 12.63
Fig. 7g	DSI – Rearing duration	two-way ANOVA	Genotype	**<0.0001******	F (1, 49) = 19.68
			Sex	0.1759	F (1, 49) = 1.886
			Genotype x sex	0.5585	F (1, 49) = 0.3470
Fig. 7h	DSI – Play behaviors duration	two-way ANOVA	Genotype	0.5041	F (1, 54) = 0.4522
			Sex	**0.0395***	F (1, 54) = 4.450
			Genotype x sex	0.5874	F (1, 54) = 0.2980
Fig. 7i	DSI – Following duration	two-way ANOVA	Genotype	0.6012	F (1, 54) = 0.2765
			Sex	0.3069	F (1, 54) = 1.064
			Genotype x sex	0.2882	F (1, 54) = 1.151
Fig. 7j	DSI – Anogenital sniffing duration	two-way ANOVA	Genotype	0.8292	F (1, 54) = 0.04699
			Sex	0.0542	F (1, 54) = 3.872
			Genotype x sex	0.6978	F (1, 54) = 0.1523
Fig. 7k	DSI – Self-grooming duration	two-way ANOVA	Genotype	0.0958	F (1, 53) = 2.876
			Sex	0.8833	F (1, 53) = 0.02176
			Genotype x sex	0.2195	F (1, 53) = 1.544

Supplementary Table S20. Statistical comparisons direct social interaction (DSI) test parameters for effects of genotype, sex, and genotype-by-sex interaction in *Magel2*^{Pmut} rats and wild-type controls (two-way ANOVA).

Figure	Parameter	Statistical test	Comparison	Adjusted P Value	t
Fig. 7f	DSI – Nose-to-nose contact duration	*post hoc* t-test with Bonferroni correction	WT vs. Pmut - Male	0.9891	0.1785
			WT vs. Pmut – Female	**<0.0001******	5.193

Supplementary Table S21. Sex-specific *post hoc* comparisons following detection of a significant genotype-by-sex interaction in direct social interaction (DSI) parameters in *Magel2*^{Pmut} rats and wild-type controls (*post hoc* unpaired, two-tailed t-tests with Bonferroni correction).

Figure	Parameter	Sample size before outlier analysis				Outliers removed (ROUT, Q = 1%)	Sample size after outlier analysis			
		WT & WT male	WT & WT female	Pmut & WT male	Pmut & WT female		WT male	WT female	Pmut male	Pmut female
Fig. S7	50-kHz USVs Number of calls	15	13	14	17	0	15	13	14	17
Fig. S7	50-kHz USVs Call length	15	13	14	17	0	15	13	14	17
Fig. S7	50-kHz USVs Tonality	15	13	14	17	0	15	13	14	17
Fig. S7	50-kHz USVs Sinuosity	15	13	14	17	0	15	13	14	17
Fig. S7	50-kHz USVs Prin. frequency	15	13	14	17	0	15	13	14	17
Fig. S7	50-kHz USVs Min. frequency	15	13	14	17	0	15	13	14	17
Fig. S7	50-kHz USVs Max. frequency	15	13	14	17	0	15	13	14	17

Supplementary Table S22. Sample size before and after outlier analysis (ROUT, Q = 1%) for 50-kHz USVs during social interaction of *Magel2*^{Pmut} rats and wild-type controls with a wild-type stimulus rat. Note: Since USV recordings always included the calls emitted by the wild-type stimulus rats, it was not possible to assign USVs to specific animals. Therefore, recordings were performed and compared in a pairwise manner with the respective wild-type stimulus rat (WT & WT or Pmut & WT). One male *Magel2*^{Pmut} and wild-type pairing was excluded from the USV analysis due to technical problems. Prin.: Principal; Min.: Minimum; Max.: Maximum.

Figure	Parameter	Statistical test	Source of variation	P Value	F (DFn, DFd)
Fig. S7	50-kHz USVs Number of calls	two-way ANOVA	Genotype	0.0586	F (1, 55) = 3.729
			Sex	**0.0272***	F (1, 55) = 5.148
			Genotype x sex	0.6070	F (1, 55) = 0.2676
Fig. S7	50-kHz USVs Call length	two-way ANOVA	Genotype	0.7509	F (1, 55) = 0.1018
			Sex	0.1132	F (1, 55) = 2.591
			Genotype x sex	0.3538	F (1, 55) = 0.8744
Fig. S7	50-kHz USVs Tonality	two-way ANOVA	Genotype	0.1158	F (1, 55) = 0.007757
			Sex	**0.0015****	F (1, 55) = 2.553
			Genotype x sex	0.9301	F (1, 55) = 11.17
Fig. S7	50-kHz USVs Sinuosity	two-way ANOVA	Genotype	0.7137	F (1, 55) = 0.1360
			Sex	**<0.0001******	F (1, 55) = 19.53
			Genotype x sex	0.6528	F (1, 55) = 0.2046
Fig. S7	50-kHz USVs Prin. frequency	two-way ANOVA	Genotype	0.0783	F (1, 55) = 3.218
			Sex	0.1473	F (1, 55) = 2.160
			Genotype x sex	0.4865	F (1, 55) = 0.4909
Fig. S7	50-kHz USVs Min. frequency	two-way ANOVA	Genotype	0.2122	F (1, 55) = 1.593
			Sex	0.6692	F (1, 55) = 0.1845
			Genotype x sex	0.8799	F (1, 55) = 0.02304
Fig. S7	50-kHz USVs Max. frequency	two-way ANOVA	Genotype	0.0526	F (1, 55) = 3.923
			Sex	0.0633	F (1, 55) = 3.593
			Genotype x sex	0.7152	F (1, 55) = 0.1345

Supplementary Table S23. Statistical comparisons of 50-kHz USVs during social interaction of *Magel2*^{Pmut} rats and wild-type controls with a wild-type stimulus rat for effects of genotype, sex, and genotype-by-sex interaction (two-way ANOVA). Prin.: Principal; Min.: Minimum; Max.: Maximum.
